# Mechanosensing by the α_6_-integrin confers an invasive fibroblast phenotype and mediates lung fibrosis

**DOI:** 10.1038/ncomms12564

**Published:** 2016-08-18

**Authors:** Huaping Chen, Jing Qu, Xiangwei Huang, Ashish Kurundkar, Lanyan Zhu, Naiheng Yang, Aida Venado, Qiang Ding, Gang Liu, Veena B. Antony, Victor J. Thannickal, Yong Zhou

**Affiliations:** 1 Department of Medicine, Division of Pulmonary, Allergy and Critical Care Medicine, University of Alabama at Birmingham, Birmingham, Alabama 35294 USA; 2 The Second Xiangya Hospital, Central-South University, Changsha 410011, China; 3 Department of Medicine, University of California at San Francisco, San Francisco, California 94143 USA

## Abstract

Matrix stiffening is a prominent feature of pulmonary fibrosis. In this study, we demonstrate that matrix stiffness regulates the ability of fibrotic lung myofibroblasts to invade the basement membrane (BM). We identify α_6_-integrin as a mechanosensing integrin subunit that mediates matrix stiffness-regulated myofibroblast invasion. Increasing α_6_-expression, specifically the B isoform (α_6_B), couples β_1_-integrin to mediate MMP-2-dependent pericellular proteolysis of BM collagen IV, leading to myofibroblast invasion. Human idiopathic pulmonary fibrosis lung myofibroblasts express high levels of α_6_-integrin *in vitro* and *in vivo*. Genetic ablation of α_6_ in collagen-expressing mesenchymal cells or pharmacological blockade of matrix stiffness-regulated α_6_-expression protects mice against bleomycin injury-induced experimental lung fibrosis. These findings suggest that α_6_-integrin is a matrix stiffness-regulated mechanosensitive molecule which confers an invasive fibroblast phenotype and mediates experimental lung fibrosis. Targeting this mechanosensing α_6_(β_1_)-integrin offers a novel anti-fibrotic strategy against lung fibrosis.

## Abstract (web summary)

Matrix stiffening is a feature of pulmonary fibrosis, and is amplified by lung myofibroblasts. Here the authors find that a6 integrin expression is upregulated on lung myofibroblasts in response to matrix stiffness, and this integrin is required for myofibroblast invasion, and fibrosis in an experimental disease model.

## Graphical abstract

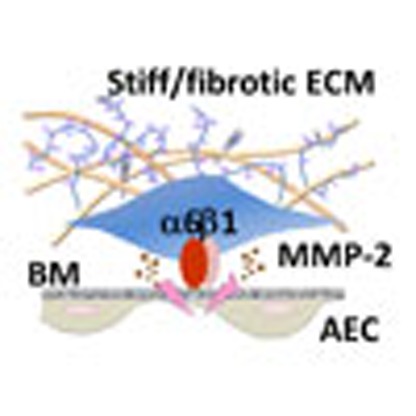

Matrix stiffening is a prominent feature of pulmonary fibrosis^1^,^2^. Accumulating evidence suggests that mechano-interactions between fibrotic lung fibroblasts (known as myofibroblasts) and stiffened, fibrotic extracellular matrix (ECM) provide a feed-forward mechanism that amplifies lung fibrosis^1^,^3^,^4^,^5^,^6^. Elucidating the key mechanosensitive molecules that confer fibrogenic properties to myofibroblasts may uncover novel therapeutic strategies for fibrotic lung diseases, including human idiopathic pulmonary fibrosis (IPF).

Fibroblasts sense the mechanical properties of the ECM by integrin and non-integrin mechanoreceptors^7^. Integrins are cell-surface heterodimers composed of non-covalently associated α- and β-transmembrane subunits. As the major force-bearing molecular links between cells and the ECM, integrins play a central role in determining how cells sense and respond to their mechanical environment. α_6_-Containing integrins serve as cellular receptors for the members of laminin family, a major structural component of the basement membrane (BM). α_6_ (ITGA6) dimerizes with either β_1_- (ITGB1) or β_4_- (ITGB4) integrin subunit to form α_6_β_1_- or α_6_β_4_-integrin complex. In normal human embryonic and adult tissues, α_6_-integrins are found to be prominently expressed in epithelia, whereas normal fibroblasts express little α_6_-integrins^8^. α_6_β_4_-Integrin is the main component of hemidesmosome and is expressed by airway epithelial cells in healthy adults^9^. Mice null for α_6_-integrins are deficient in hemidesmosome formation and die at birth with severe blistering of skin and other epithelia^10^. A recent study showed that α_6_β_4_ marks a subpopulation of lung epithelial progenitor cells which are capable of self-renewal and differentiation into multiple respiratory epithelial cell types^11^. Although the role of α_6_-integrins in fibroblasts are limited, increasing α_6_-integrin expression has been observed in transformed neoplastic fibroblasts in human metastatic fibrosarcoma^12^.

The BM is a dense, sheet-like structure at the interface of epithelial/endothelial and mesenchymal tissues. It maintains the polarity of lung epithelial cells and provides a physical barrier between lung epithelium and the mesenchyme. Cells invade the BM by adhering to BM matrices and engaging proteinase-dependent dissolution of BM at focal adhesions^13^. Alternatively, cells may transmigrate through the BM by proteinase-independent disassembly of BM superstructure and enlargement of pore size, a process termed mesenchymal–amoeboid transition^14^.

The integrity of the BM maintains a healthy lung epithelium and its integrity is essential for restoration of alveolar epithelial homoeostasis following lung injury^15^. Loss of the BM integrity has been observed in IPF^16^. Mechanisms underlying disruption of the BM integrity in IPF are currently not well understood. Disruption of alveolar BMs prevents an orderly repair of the damaged alveolar type I epithelial cells, thus impairing normal reepithelialization^17^. It has been observed that intact BMs suppress programmed cell death in mammary epithelium and other tissues^18^,^19^, suggesting that loss of the BM integrity may also promote alveolar epithelial cell apoptosis.

Fibrotic lung fibroblasts are characterized by an invasive phenotype^20^,^21^,^22^,^23^,^24^. White *et al*.^20^ reported that constitutively lower levels of PTEN in IPF lung myofibroblasts promote cell invasion into the BM, whereas α_4_β_1_ ligation-dependent expression of PTEN prevents cell invasion. Li *et al*.^21^ found that mouse lung myofibroblasts isolated from hyaluronan synthase 2 transgenic mice acquire an invasive phenotype. In a recent study, Lovgren *et al*.^22^ showed that knockdown of β-arrestin2 in IPF lung myofibroblasts attenuates cell invasiveness. Proteinases capable of degrading the BM include MMP2 and MMP9 of type IV collagenases. In the fibroblastic foci of IPF, subepithelial myofibroblasts close to the areas of BM disruption express MMP-2 as well as MMP-9 (ref. 25), suggesting that MMPs may mediate proteinase-dependent IPF myofibroblast invasion into the BM and the disruption of BM integrity.

In this study, we report that α_6_ is a matrix stiffness-regulated mechanosensitive integrin subunit. Stiff matrix-induced upregulation of α_6_-expression mediates IPF lung myofibroblast invasion into the BM. We explore mechanotransductive mechanisms for α_6_-integrin expression and demonstrate that the expression of this integrin subunit by lung myofibroblasts is increased in both human IPF and bleomycin injury-induced experimental lung fibrosis. Animal studies using genetic and pharmacological approaches support targeting mechanosensitive α_6_-integrin as a novel therapeutic strategy for lung fibrosis.

## Results

### α_6_ Is a matrix stiffness-regulated mechanosensitive gene

**Figure 1:**
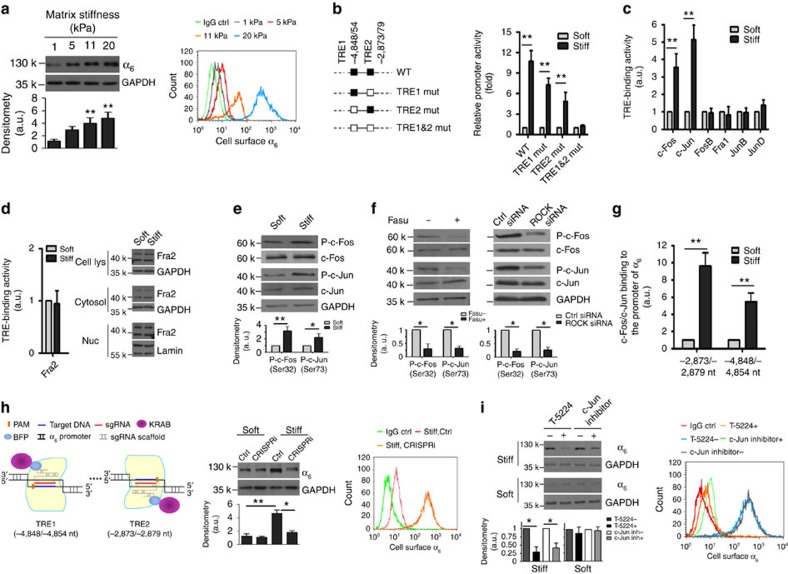
Stiff matrix upregulates α_6_-expression by ROCK-dependent activation of c-Fos/c-Jun transcription complex. (**a**) IPF lung myofibroblasts were cultured on PA hydrogels with increasing stiffness (1, 5, 11 and 20 kPa). Levels of α_6_-protein were determined by immunoblot and flow cytometry, respectively. In flow cytometry, non-immune rat IgG2a, κ was used as isotype IgG control. (**b**) Schematic shows the WT and mutated human α_6_-promoters. Promoter activities were determined by luciferase assay. (**c**) Nuclear extracts from myofibroblasts cultured on soft and stiff matrix were incubated with immobilized oligonucleotides containing TREs. The TRE-binding activities of six AP-1 components as indicated were quantified by colorimetric enzyme-linked immunosorbant assay (ELISA). (**d**) The TRE-binding activity of Fra2 in nuclear extracts was quantified by colorimetric ELISA. Levels of Fra2 protein in cell lyates, cytoplasmic and nuclear fractions were determined by immunoblot. (**e**) Protein levels of phospho and total c-Fos and c-Jun under soft versus stiff matrix conditions were determined by immunoblot. (**f**) Effects of ROCK inhibitor Fasudil (Fasu) and ROCK-specific siRNAs on stiff matrix-induced phosphorylation of c-Fos and c-Jun. (**g**) The binding of c-Fos/c-Jun complex to the α_6_-promoter under soft versus stiff matrix conditions was measured by quantitative chromatin immunoprecipitation. (**h**) Schematic shows sgRNA-mediated targeted expression of KRAB transcription repressor at the distal TRE1 and the proximal TRE2 regions in human α_6_**-**promoter. Effects of CRISPRi-based disruption of c-Fos/c-Jun-dependent promoter activation on stiff matrix-induced α_6_**-**expression were evaluated by immunoblot and flow cytometry analyses. Control (Ctrl) indicates cells transfected with empty vector. (**i**) Effects of c-Fos/c-Jun inhibitors (T-5224 and c-Jun peptides) on matrix stiffness-regulated α_6_-expression were evaluated by immunoblot and flow cytometry. Results are the means ±s.d. of at least three separate experiments; *P<0.05; **P<0.01; one-way analysis of variance. a.u., arbitrary units.

To determine whether matrix stiffness regulates the expression of cell adhesion and ECM molecules, we performed a qPCR array analysis that contains 84 genes, including 16 integrin subunits in primary lung myofibroblasts isolated from patients with IPF (Supplementary Fig. 1). We found that 10 genes were increased or decreased ⩾twofold under stiff versus soft matrix conditions; the differential mRNA expression of 7 genes was statistically significant (Table 1). The α_6_-integrin subunit mRNA was increased 5.3-fold on stiff matrix. To validate these gene expression data, we performed additional studies at the protein level to determine if the α_6_-subunit is regulated by matrix stiffness; we observed a matrix stiffness grade-dependent increase in α_6_-integrin expression when cells were grown on polyacrylamide (PA) gels with stiffness ranging from 1 to 20 kPa (Fig. 1a). Similar results were obtained when cells were grown on a second stiffness-tunable substrate system of polydimethylsiloxane hydrogels (Supplementary Fig. 2a). Lung myofibroblasts isolated from bleomycin-treated mice also respond to matrix stiffening with increased α_6_-expression (Supplementary Fig. 2b,c). These results identify, for the first time, the α_6_-integrin subunit as a matrix stiffness-regulated mechanosensitive gene/protein.

A bioinformatics search identified AP-1-specific TPA-response elements (TREs) (TGA(G/C)TCA) in the promoter region of human and mouse α_6_-integrin genes (Supplementary Fig. 2d). It has been shown that mechanical stretch activates AP-1 in human osteoblastic cells^26^. Activation of AP-1 transcription complex is associated with cancer cell invasion^27^,^28^. On the basis of this information, we sought to determine whether the AP-1 transcription complex mediates stiff matrix-induced α_6_-integrin gene expression. We first determined whether matrix stiffness regulates the promoter activity of α_6_-gene. A 6,200-bp of wild-type (WT) human proximal α_6_-promoter reporter and 3 mutated promoter reporters harbouring mutations at the specific AP-1-binding DNA sequences, TRE1 (−4,848 to −4,854 nt), TRE2 (−2,873 to −2,879 nt) or both TRE1 and TRE2, were transfected into IPF lung myofibroblasts (Fig. 1b). In cells transfected with WT α_6_-promoter reporter, stiff matrix significantly increased luciferase expression (Fig. 1b), suggesting that human α_6_-promoter activity is enhanced by stiff matrix. Deletion of either TRE1 or TRE2 inhibited stiff matrix-induced increases in α_6_-promoter activity. Deletion of both TRE1 and TRE2 completely blocked stiff matrix-induced α_6_-promoter activation (Fig. 1b). Altogether, these data suggest that stiff matrix activates α_6_-promoter by an AP-1-dependent mechanism.

Next, we investigated effects of matrix stiffness on the binding of seven major AP-1 components (c-Fos, c-Jun, FosB, Fra1, Fra2, JunB and JunD) to immobilized TREs. Stiff matrix selectively increased c-Fos and c-Jun binding to immobilized oligonucleotides containing TREs, whereas the binding of FosB, Fra1, JunB and JunD to TREs were not altered by matrix stiffness (Fig. 1c). Previous studies have shown that Fos-related protein Fra2 is associated with human IPF and spontaneous development of lung fibrosis in mice^29^. In our studies, neither the binding of Fra2 to immobilized TREs nor its expression or cytoplasmic/nuclear distribution were regulated by matrix stiffness (Fig. 1d), suggesting that matrix stiffness is unlikely to regulate Fra2 activity. It has been shown that phosphorylation of c-Fos at Ser32/Thr232 and c-Jun at Ser63/Ser73 is associated with increased DNA-binding activity of c-Fos and c-Jun^30^. We found that stiff matrix (20 kPa) in comparison with soft matrix (1 kPa) increased the levels of phospho c-Fos at Ser32 and phospho c-Jun at Ser73 (Fig. 1e); the total protein and the mRNA levels of c-Fos and c-Jun were not altered by matrix stiffness (Fig. 1e and Supplementary Fig. 2e). Previously, we have shown that stiff matrix activates protein serine/threonine kinase ROCK in lung myofibroblasts^4^. Here, we observed that inhibition of ROCK by fasudil or siRNA-based knockdown blocked stiff matrix-induced c-Fos and c-Jun phosphorylation (Fig. 1f), suggesting that ROCK mediates stiff matrix-induced phosphorylation and activation of c-Fos/c-Jun transcription complex. Quantitative chromatin immunoprecipitation assay demonstrated that stiff matrix significantly increased the constitutive enrichment of α_6_-promoter DNA in phospho c-Fos antibody-immunoprecipitated chromatin of IPF lung myofibroblasts at both the proximal (−2,873/−2,879 nt) and distal (−4,848/−4,854 nt) TRE sites (Fig. 1g). Altogether, these data suggest that the c-Fos/c-Jun complex of AP-1 transcription factor family mediates stiff matrix-dependent transactivation of α_6_-gene.

To determine whether inhibition of c-Fos/c-Jun-dependent α_6_-promoter activation blocks stiff matrix-induced α_6_-expression, we used CRISPR interference (CRISPRi) technology, which allows sequence-specific disruption of transcription factor binding to the promoter for gene silencing^31^. Two single guide RNAs (sgRNAs) were designed to specifically bind to a 20-bp DNA sequence next to each of two TREs in human α_6_-promoter (Fig. 1h). Expression of deactivated Cas9 (dCas9)-KRAB fusion proteins, in which dCas9 provides a DNA-binding platform at sites defined by sgRNAs for KRAB domain-mediated repression of c-Fos/c-Jun-dependent α_6_-promoter activation, blocked stiff matrix-induced α_6_-expression (Fig. 1h). Similar to CRISPRi, pharmacologic inhibition of c-Fos/c-Jun activity by T-5224, a selective c-Fos/AP-1 inhibitor, or by c-Jun peptide inhibitor also blocked stiff matrix-induced α_6_-integrin expression (Fig. 1i).

We also observed that stiff matrix increases c-fos and c-jun binding to immobilized TREs in mouse lung myofibroblasts, whereas the binding of fosB, fra1, fra2, junB and junD to TREs were not altered by matrix stiffness (Supplementary Fig. 2f). Quantitative chromatin immunoprecipitation assay demonstrated that stiff matrix significantly increased the enrichment of mouse α_6_-promoter DNA in phospho c-Fos antibody-immunoprecipitated chromatin of mouse lung myofibroblasts (Supplementary Fig. 2g). Pharmacologic inhibition of c-Fos/c-Jun activity by T-5224 or decoy oligodeoxynucleotides^32^ blocked stiff matrix-induced mouse α_6_-expression (Supplementary Fig. 2h). Taken together, these data support a role for the c-Fos/c-Jun-dependent mechanotransduction pathway in stiff matrix-induced α_6_-expression.

### α_6_ Mediates lung myofibroblast invasion

**Figure 2:**
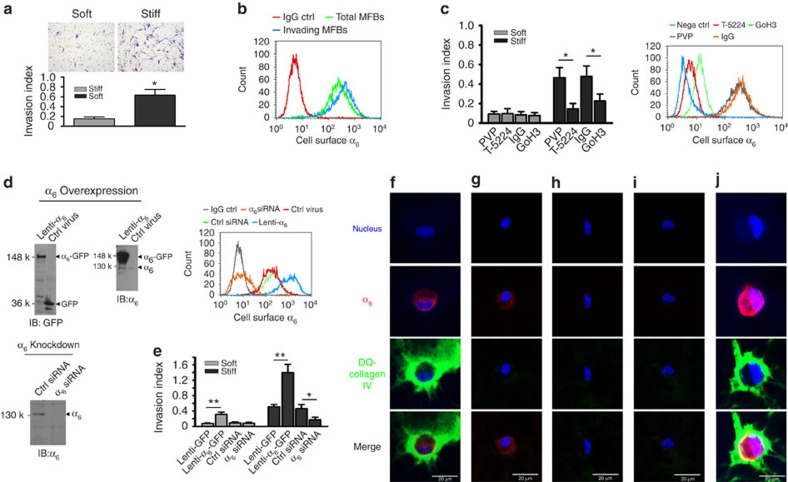
α_6_ Mediates matrix stiffness-dependent lung myofibroblast invasion into the BM. (**a**) The ability of IPF myofibroblasts cultured on soft versus stiff matrix to invade the BM was evaluated by Matrigel invasion assay. (**b**) α_6_-Expression on the cell surface of invading myofibroblasts versus total (myo)fibroblasts was evaluated by flow cytometry. (**c**) Effects of NKI-GoH3 and T-5224 on stiffness-regulated myofibroblast invasion into the BM. α_6_-Expression on the cell surface was evaluated by flow cytometry using FITC-labelled GoH3. PVP, a vehicle for T-5224; IgG, FITC-labelled isotype control IgG for NKI-GoH3; Nega ctrl, plain cells with no treatments and no incubation with FITC-labelled GoH3/IgG. (**d**) Overexpression of α_6_-GFP fusion protein by lentivirus and knockdown of α_6_ by siRNA in cell lysates were determined by immunoblot and flow cytometry. (**e**) Effects of overexpression or knockdown of α_6_ on stiffness-regulated myofibroblast invasion into the BM. (**f**–**j**) α_6_-expression (red) and proteolytic activation of DQ-collagen IV (green) in the absence (**f**) or presence of NKI-GoH3 (**g**), T-5224 (**h**), α_6_-siRNA (**i**) and Lenti-α_6_ (**j**) were determined by confocal immunofluorescent microscopy. Nuclei (blue) were stained by DAPI. Results are the means±s.d. of at least three separate experiments; *P<0.05, **P<0.01; one-way analysis of variance. Scale bar, 20 μm.

Fibrotic lung myofibroblasts isolated from patients with IPF are characterized by an invasive phenotype^20^,^21^,^22^,^23^,^24^. To determine whether the mechanical properties of the ECM may regulate the ability of IPF lung myofibroblasts to invade the BM, we pre-cultured primary lung myofibroblasts isolated from patients with IPF on soft (1 kPa) and stiff (20 kPa) PA hydrogel substrates. The stiffness grades of soft and stiff PA gels were within the physiologic stiffness ranges of normal and fibrotic lungs^1^,^2^. Lung myofibroblasts adapted to soft and stiff matrix were trypsinized and transferred to the invasion chambers containing BM matrices (Matrigel). We observed that cells derived from stiff PA gels had a significantly higher invasion index than cells derived from soft PA gels (Fig. 2a). Similar findings were observed when cells were cultured on soft (2 kPa) and stiff (30 kPa) polydimethylsiloxane hydrogels (Supplementary Fig. 3a). These data suggest that stiff matrix promotes IPF lung myofibroblasts to invade the BM. To confirm these findings, we designed a ‘sandwich' invasion assay in which cells cultured on soft and stiff PA gels were directly transferred to invasion chambers with the apical (dorsal) side of cells in close contact with the BM matrices (Supplementary Fig. 3b). We observed enhanced invasive properties of lung myofibroblasts on stiff matrices using this second approach (Supplementary Fig. 3c). Since Matrigel may not fully replicate BM matrices found *in vivo*, we isolated rat mesenteric BM that has been used to study cancer cell invasion^33^ to determine effects of matrix stiffness on the ability of IPF lung myofibroblasts to invade biological BMs (Supplementary Fig. 3d). We observed that IPF lung myofibroblasts pre-cultured on stiff PA gels had a higher invasive index than cells pre-cultured on soft PA gels (Supplementary Fig. 3e). Altogether, these findings indicate that matrix stiffness confers an invasive property to IPF lung myofibroblasts, specifically through the BM.

α_6_-Integrin is a major cellular receptor for laminin, a protein component of BMs. Next, we determined whether the mechanosensing α_6_-integrin regulates stiff matrix-induced lung myofibroblast invasion into the BM. We first compared the levels of α_6_-integrin on the cell surface in the subpopulation of IPF lung myofibroblasts, that is, myofibroblasts that penetrated into the BM in comparison with the total population of IPF lung myofibroblasts. Flow cytometry analysis demonstrated a higher expression of α_6_ on the cell surface of invading lung myofibroblasts relative to the total lung myofibroblast population (Fig. 2b). When lung myofibroblasts were pre-treated with NKI-GoH3 (a specific antibody that blocks α_6_-mediated cell adhesion) or T-5224 (an inhibitor of c-Fos), stiff matrix-dependent lung myofibroblast invasion into the BM was significantly inhibited (Fig. 2c). In these experiments, flow cytometry analysis demonstrated that treatment with T-5224 and GoH3 inhibited α_6_-integrin on the cell surface (Fig. 2c). Vehicle controls (IgG isotype control antibody for GoH3; polyvinylpyrrolidone (PVP) for T-5224) had no effects on lung myofibroblast invasion and α_6_-expression on the cell surface. To further determine the role of α_6_-integrin in matrix stiffness-regulated lung myofibroblast invasion into the BM, we generated lung myofibroblasts that overexpress α_6_-GFP fusion proteins or GFP alone with a lentiviral vector-based approach; an siRNA-based approach was utilized to generate lung myofibroblasts deficient in α_6_-integrin expression (Fig. 2d). Overexpression of α_6_ significantly enhanced stiff matrix-induced IPF lung myofibroblast invasion into the BM, whereas knockdown of α_6_ significantly inhibited lung myofibroblast invasion (Fig. 2e). In addition, overexpression of α_6_ was sufficient to induce BM invasion of lung myofibroblasts cultured on soft matrices. GFP control lentiviruses and control siRNA had no effects on matrix stiffness-regulated myofibroblast invasion into the BM (Fig. 2e). Altogether, these loss- and gain-of-function studies support a key role for the mechanosensitive α_6_-integrin in mediating matrix stiffness-regulated IPF lung myofibroblast invasion into the BM.

Proteolytic degradation of the BM proteins is critical for cellular invasion into the BM^34^. Next, we determined whether the α_6_-integrin mediates proteolysis of collagen IV, a major component of the BM, using fluorescent dye-quenched (DQ)-collagen IV which is quenched in its native form and emits strong fluorescence on proteolytic hydrolysis^35^. Confocal immunofluorecent microscopy showed that IPF lung myofibroblasts derived from stiff matrix expressed α_6_-integrin subunit; fluorescent signals from DQ-collagen IV were observed in the periphery of α_6_-positive lung myofibroblasts, indicative of pericellular proteolysis of collagen IV in the BM (Fig. 2f). Blocking α_6_-mediated cell adhesion with NKI-GoH3 antibody (Fig. 2g), inhibition of mechano-induction of α_6_-expression by T-5224 (Fig. 2h), and knockdown of α_6_-expression with α_6_-specific siRNA (Fig. 2i) inhibited pericellular proteolysis of collagen IV. Overexpression of α_6_-integrin enhanced pericellular proteolysis of collagen IV (Fig. 2j). The IgG isotype control antibody, PVP and scrambled control siRNA had no effects on proteolysis of collagen IV (Supplementary Fig. 4).

The matrix metalloproteinases (MMPs), MMP-2 and MMP-9, are known to degrade collagen IV^36^. In this study, we observed that stiff matrix induced an average of sixfold increases in MMP-9 mRNA as compared with soft matrix (Table 1), whereas MMP-2 mRNA expression was not altered by matrix stiffness. However, a direct comparison of relative mRNA expression in IPF lung myofibroblasts showed that the baseline MMP-2 was >20,000-fold higher than MMP-9 (Supplementary Fig. 5a). Zymographic analysis detected MMP-2 activities in both the conditioned media and cell lysates collected from IPF lung myofibroblasts cultured on soft and stiff matrix, whereas MMP-9 activities were undetectable (Supplementary Fig. 5b). Consistent with the qPCR findings, MMP-2 activities were not altered by matrix stiffness. These data suggest that IPF lung myofibroblasts primarily express MMP-2 of type IV collagenases. Next, we investigated whether MMP-2 is involved in α_6_-mediated collagen IV degradation. Knockdown of MMP-2 by siRNA blocked pericellular proteolysis of DQ-collagen IV (Supplementary Fig. 5c) and IPF lung myofibroblast invasion into BM matrices (Supplementary Fig. 5d). Collectively, these data suggest that α_6_-dependent invasion of IPF lung myofibroblasts requires pericellular proteolysis of BM collagen IV by MMP-2.

### α_6_-Expression is upregulated in lung myofibroblasts

**Figure 3:**
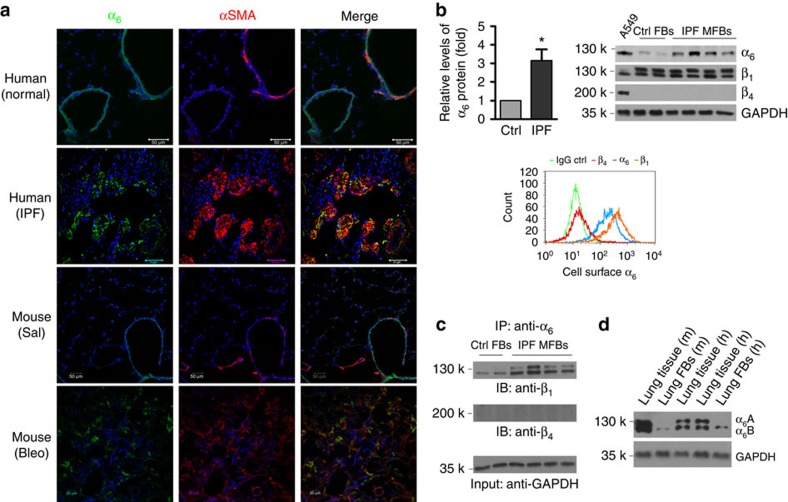
Lung myofibroblasts demonstrate increased α6-expression. (**a**) Frozen lung tissue sections obtained from failed normal human donors, patients with IPF, saline-treated mice and bleomycin-treated mice were double-stained for α_6_ (green) and αSMA (red). Nuclei were stained by DAPI (blue). Confocal immunofluorescent images were overlaid to show α_6_-expression in αSMA-positive lung myofibroblasts. Scale bar, 50 μm; scale bar, 20 μm for mouse with bleo images. (**b**) Comparison for α_6_-expression in lung (myo)fibroblasts isolated from patients with IPF (*n*=10) and non-ILD control human subjects (*n*=6) by immunoblot; Relative levels of α_6_-protein normalized to GAPDH expression. Results are the means±s.d. Representative blots for α_6_-expression as well as β_1_- and β_4_-expression were shown. A549 cells were used as positive control for β_4_-expression in immunoblot analysis. Relative levels of α_6_-, β_1_- and β_4_-expression on the cell surface of IPF lung myofibroblasts were analysed by flow cytometry. (**c**) Detection of α_6_β_1_- and α_6_β_4_-complexes in IPF lung myofibroblasts by immunoprecipitation and immunoblot. (**d**) Identification of α_6_A and α_6_B expression in human and mouse lung tissues and fibroblasts by immunoblot; *P<0.05, one-way analysis of variance.

Next, we determined whether myofibroblast expression of α_6_ is altered in a human fibrotic disorder, IPF, and in a murine model of experimental lung fibrosis. Confocal immunofluorescent microscopy demonstrated high levels of α_6_-expression in αSMA-positive lung myofibroblasts in fibroblastic foci of lung tissues of human subjects with IPF, as well as in fibrotic lesions following bleomycin lung injury in mice; in contrast, α_6_-expression was primarily observed in the airway epithelium of normal human and mouse lungs (Fig. 3a). Primary lung myofibroblasts isolated from human subjects with IPF expressed significantly higher levels of α_6_ than primary lung fibroblasts isolated from control subjects (Fig. 3b,c). α_6_-Integrin contains two structural variants, α_6_A and α_6_B, owing to alternatively spliced transcripts^37^. In addition, α_6_-subunit pairs with either the β_1_- or β_4_-subunit to form functional integrin complexes. We demonstrated that although both α_6_A and α_6_B were expressed in human and mouse lung tissues at equivalent levels, lung (myo)fibroblasts primarily express the shorter α_6_B isoform (Fig. 3d). Interestingly, both normal lung fibroblasts and fibrotic lung myofibroblasts express similar levels of the β_1_-integrin subunit, while β_4_-protein expression in lung (myo)fibroblasts is not detectable (Fig. 3b,c). These results indicate that, in the context of fibrotic lung injury *in vivo* both in mice and humans, lung myofibroblasts express high levels of the α_6_-integrin; the α_6_Bβ_1_ is the primary α_6_-integrin complex expressed by lung (myo)fibroblasts.

### Inhibition of α_6_ protects against experimental lung fibrosis

**Figure 4:**
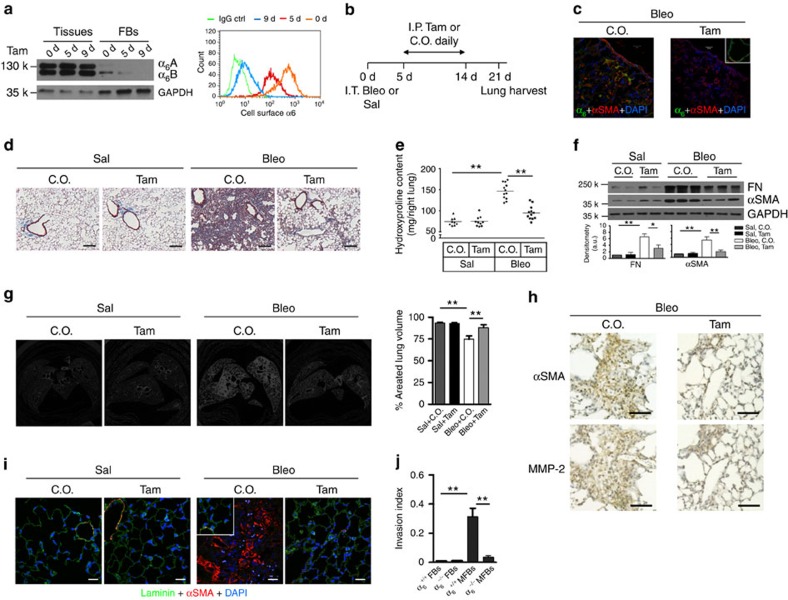
Fibroblast-specific deletion of α_6_ protects mice against bleomycin injury-induced experimental lung fibrosis. (**a**) Time-dependent deletion of α_6_-expression in lung fibroblasts in conditional α_6_^−/−^ mice following tamoxifen (Tam) administration. Levels of α_6_-protein in cell lysates and on the cell surface were determined by immunoblot and flow cytometry. (**b**) Schematic shows the design of animal experiments. (**c**) Frozen lung tissue sections from bleomycin-treated mice were double-stained for α_6_ (green) and αSMA (red). Nuclei were stained by DAPI (blue). Confocal immunofluorescent images were overlaid to show α_6_-expression in αSMA-positive lung myofibroblasts. Epithelial α_6_-expression in tam-treated mice was shown in the inset. Scale bar, 20 μm. (**d**) Representative images for trichrome staining of collagens in paraffin-embedded lung tissue sections. Scale bar, 150 μm. (**e**) Quantification of hydroxyproline contents in right lungs of mice from four mouse groups: Sal+C.O., Sal+Tam, Bleo+C.O. and Bleo+Tam. (**f**) Quantification of fibronectin (FN) and αSMA protein expression in left lungs by immunoblot. Shown are representative blots. (**g**) Shown are representative images for *ex vivo* mid-lung transaxial μCT scans. The average percentages of aerated lung volumes of mice in four groups (*n*=5 per group) are shown in the bar graph. (**h**) Immunohistochemical staining of two adjacent lung sections shows Mmp-2 expression in the areas of αSMA-expressing lung myofibroblasts. Nuclei were stained by hematoxylin (blue). Scale bar, 100 μm. (**i**) Frozen lung tissue sections were stained for laminin (green) (a component of the BMs) and αSMA (red). Nuclei were stained by DAPI (blue). Inset shows laminin and αSMA staining in the relatively normal area of the same lung section. Scale bar, 20 μm. (**j**) Lung (myo)fibroblasts (FB and MFB) were isolated from mice in four groups. The ability of (M)FBs to invade the BM matrices was determined by invasion assay. Results are the means±s.d. of three separate experiments, each performed in triplicates; *P<0.05 and **P<0.01; one-way analysis of variance. Bleo, bleomycin; C.O., corn oil; Sal, saline.

To determine whether α_6_-expression in lung myofibroblasts plays a causal role in lung fibrogenesis, we generated conditional α_6_-knockout (α_6_-CKO) mice in which α_6_-gene is specifically deleted in collagen I-producing cells by intraperitoneal injection of tamoxifen. In pilot studies, we confirmed that tamoxifen treatment induces a time-dependent deletion of α_6_-expression in mouse lung fibroblasts (Fig. 4a). Almost complete deletion of α_6_-expression was observed after treatment of tamoxifen for 9 consecutive days. No significant reduction of α_6_-expression was observed in mouse whole-lung homogenates, suggesting that α_6_ deletion was mesenchymal cell-specific (Fig. 4a). Consistent with our previous findings (Fig. 3d), we observed that primary lung fibroblasts isolated from mice primarily express α_6_B isoform. On the basis of these time-course studies, we designed our experimental procedures as depicted in Fig. 4b: α_6_-CKO mice were given intratracheal bleomycin or saline on day 0. Since bleomycin-induced mouse lung fibrosis is characterized by acute lung injury and inflammation in the early phase (day 0–10) followed predominantly by lung fibrosis (day >14), we started intraperitoneal tamoxifen or corn oil (vehicle control for tamoxifen) treatment on day 5 post-bleomycin administration so that a complete knockout of α_6_ in lung fibroblasts would be expected to occur at ∼14 days after lung injury; this minimizes potential effects of α_6_ deletion on the early phases of lung injury and inflammation. Mouse lungs were collected at day 21 and evaluated for lung fibrosis. Confocal immunofluorescent microscopy confirmed that αSMA-positive lung myofibroblasts in corn oil-treated control mice expressed α_6_-integrin, whereas lung myofibroblasts in tamoxifen-treated mice did not (Fig. 4c). Mice with conditional deletion of the α_6_-gene during the post-inflammatory fibrotic phase of lung repair demonstrated marked attenuation of fibrotic responses, as assessed by trichrome staining of the lung for collagen (Fig. 4d), whole-lung hydroxyproline content (Fig. 4e), protein levels of fibronectin and αSMA in whole-lung homogenates (Fig. 4f), and micro-CT-based measurements of aerated lung volume, an inverse surrogate marker for pulmonary fibrosis^38^ (Fig. 4g). In addition, Mmp-2 expression was found in the area of αSMA-expressing lung myofibroblasts in both corn oil-treated control mice and tamoxifen-treated α_6_-CKO mice (Fig. 4h). Saline-treated WT and α_6_-CKO mice and bleomycin-treated α_6_-CKO mice showed intact continuous BMs, as demonstrated by immunostain of the BM component laminin. In contrast, the BM signals were largely disrupted in myofibroblast-enriched fibrotic regions of lungs from bleomycin-treated WT mice (Fig. 4i). Primary lung myofibroblasts isolated from bleomycin-treated α_6_-CKO mice (α_6_^−/−^ MFBs) demonstrated reduced capacity for BM invasion as compared with primary lung myofibroblasts isolated from bleomycin-treated WT mice (α_6_^+/+^ MFBs); primary lung fibroblasts isolated from saline-treated α_6_-CKO mice (α_6_^−/−^ FBs) and WT mice (α_6_^+/+^ FBs) showed minimal invasion into the BM (Fig. 4j).

**Figure 5:**
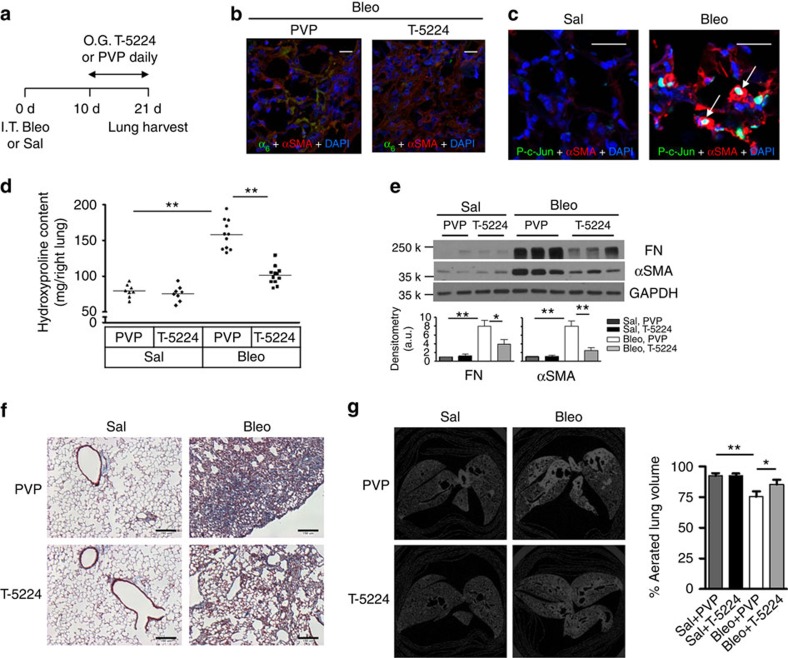
Pharmacological inhibition of c-Fos/c-Jun protects mice against bleomycin injury-induced experimental lung fibrosis. (**a**) Animal experimental design. (**b**) Overlaid confocal immunofluorescent images show α_6_-expression (green) in αSMA-positive lung myofibroblasts (red) in mice with treatments as indicated. Nuclei were stained by DAPI (blue). Scale bar, 20 μm. (**c**) Overlaid confocal immunofluorescent images show phospho c-Jun (green) in the nuclei of αSMA-positive lung myofibroblasts (red) (arrows) in mice treated with saline or bleomycin. Nuclei were stained by DAPI (blue). Scale bar, 20 μm. (**d**) Quantification of hydroxyproline contents in right lungs of C57BL6 mice in four groups: Sal+PVP, Sal+T-5224, Bleo+PVP and Bleo+T-5224. Results are the means ±s.d. (**e**) Quantification of FN and αSMA protein expression in left lungs by immunoblot. Shown are representative blots. (**f**) Representative images for trichrome staining of collagens in paraffin-embedded lung tissue sections. Scale bar, 150 μm. (**g**) Shown are representative images for *ex vivo* mid-lung transaxial μCT scans. The average percentages of aerated lung volumes are shown in the bar graph (*n*=5 mice per group). Results are the means±s.d.; *P<0.05 and **P<0.01; one-way analysis of variance. O.G., oral gavage.

Since stiff matrix upregulates α_6_-expression through a c-Fos/c-Jun-dependent mechanotransduction pathway (Fig. 1), we determined whether pharmacological blockade of c-Fos/c-Jun pathway protects WT C57BL6 mice against bleomycin injury-induced experimental lung fibrosis. To minimize the potential effects of T-5224 on lung injury and inflammation, we started T-5224 or PVP (vehicle control) treatment at day 10 post-bleomycin administration (Fig. 5a). Mice treated with vehicle control showed α_6_-expression in αSMA-expressing lung myofibroblasts, whereas α_6_-expression in lung myofibroblasts was greatly reduced in mice treated with T-5224 (Fig. 5b). In mice treated with bleomycin, phospho c-Jun was observed in the nuclei of αSMA-positive lung myofibroblasts (Fig. 5c). In contrast, phospho c-Jun was absent in the lungs of saline-treated control mice. These data suggest that c-Fos/c-Jun signalling is activated in mouse lung fibrosis. Similar to genetic ablation of α_6_ in lung mesenchymal cells, we observed that administration of T-5224 during the post-inflammatory fibrotic phase abrogated bleomycin injury-induced experimental lung fibrosis in mice (Fig. 5d, hydroxyproline content; Fig. 5e, immunoblot for fibronectin and α-SMA; Fig. 5f, Masson's trichrome staining; Fig. 5g, micro-CT analysis of aerated lung volume). Control studies showed that tamoxifen had no effect on bleomycin-induced lung fibrosis in *Itga6* floxed mice (Supplementary Fig. 6a,b). Quantification of inflammatory cells in bronchoalveolar lavage on day 14 demonstrated that post-inflammatory deletion of α_6_ in mesenchymal cells or T-5224 treatment did not alter the inflammatory response to bleomycin lung injury (Supplementary Fig. 6c,d). Immunostaining of nuclear Ki-67, a cell proliferation marker, revealed that the vast majority of αSMA-positive lung myofibroblasts were non-proliferative (Supplementary Fig. 6e). Neither α_6_ deletion nor T-5224 treatment altered the proliferative rate of lung myofibroblasts. Altogether, these results provide strong support for a critical pro-fibrotic role for the mechanosensitive α_6_-integrin subunit, at least in part, by its capacity to mediate myofibroblast invasion.

## Discussion

**Figure 6:**
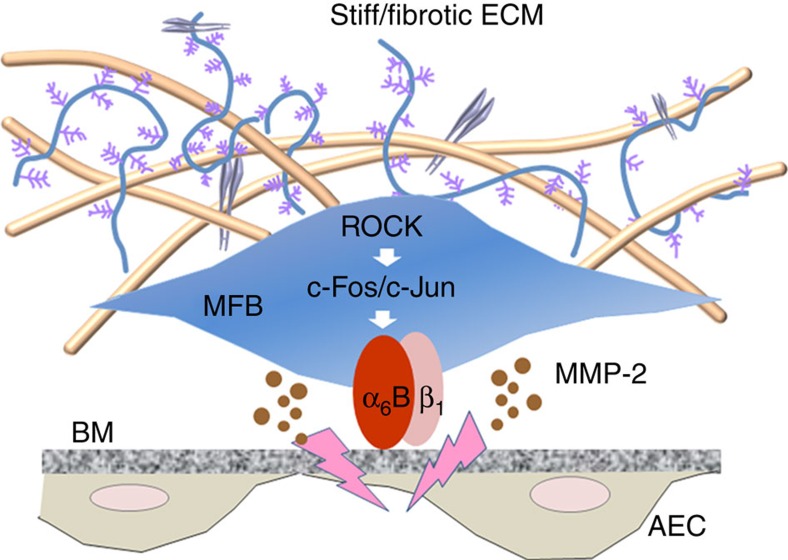
A model for mechanosensing α_6_ in the regulation of lung myofibroblast invasion into the BM. Stiff/fibrotic matrix upregulates α_6_-expression by ROCK-dependent activation of c-Fos/c-Jun transcription complex. Interactions between α_6_-integrins, specifically α_6_Bβ_1_-integrins, and the BM bring lung myofibroblasts into the close proximity to the BM. This facilitates MMP-2-mediated pericellular proteolysis of BM component collagen IV, leading to lung myofibroblast invasion.

In this study, we identified that the α_6_-integrin subunit is a matrix stiffness-regulated mechanosensitive protein. Stiff matrix upregulates α_6_-integrin expression by ROCK-dependent activation of a c-Fos/c-Jun transcription complex in fibroblasts. Increased expression of α_6_-integrin is associated with enhanced capacity for lung myofibroblast invasion into the BM. We predict that α_6_-mediated myofibroblast-BM interactions bring myofibroblasts into the close proximity to the BM, which facilitates MMP-2-dependent pericellular proteolysis of collagen IV in the BM, thus promoting myofibroblast invasion (Fig. 6). Furthermore, we show that genetic deletion of α_6_ in (myo)fibroblasts or pharmacological blockade of the c-Fos/c-Jun mechanotransduction pathway, which regulates α_6_-expression, protects mice against experimental lung fibrosis. These *in vivo* studies suggest that targeting mechanosensing α_6_-integrins, specifically α_6_Bβ_1_, may provide a novel anti-fibrotic strategy against pulmonary fibrosis. Previous studies have shown that mechanosensing by integrins may involve unmasking of cryptic sites within the cytoplasmic domains that allow for the binding of signalling molecules and/or transition of integrins from low- to high-affinity binding states^39^. The present study, along with that of others^40^,^41^, suggests that regulation of integrin expression *per se* is an important mechanism for integrin-mediated mechanosensing.

We observe that α_6_-expression is increased in lung myofibroblasts of human IPF and bleomycin injury-induced lung fibrosis in mice. It has been reported that in IPF, lung epithelial cells express high levels of laminins adjacent to fibroblast foci^42^. This finding is consistent with our observations that interactions between stiff matrix-regulated α_6_ in lung myofibroblasts and the BM mediate IPF myofibroblast invasion. Interestingly, BM-associated laminin-5 is associated with stromal fibroblastic reaction at the invasive front of lung adenocarcinoma, which may facilitate its invasiveness^43^. In addition, human prostate cancer cells express high levels of α_6_-integrins; α_6_β_1_-integrins mediate prostate cancer metastasis to laminin-rich bone microenvironment^44^. α_6_-Integrins also regulate the invasive phenotype of HT 1080 fibrosarcoma cells^45^, and the levels of α_6_-integrins correlate with the degree of tumorigenicity of human neoplastic fibroblasts^12^. In addition to the regulation of cell invasion, there is accumulating evidence that α_6_β_1_-integrins promote cell survival through both PI3K/Akt-dependent and -independent pathways^46^,^47^. It has been reported that α_6_β_1_-integrins mediate collagen deposition in gingival fibroblasts^48^, although the underlying mechanisms remain to be determined. Thus, it is possible that stiff matrix-induced α_6_-expression may not only regulate lung myofibroblast invasion, but contribute to their anti-apoptotic and matrix-remodelling properties as well.

We previously demonstrated that matrix stiffening activates RhoA/ROCK mechanosensitive signal pathway in lung (myo)fibroblasts^4^. In this study, we showed that stiff matrix-induced phosphorylation of c-Jun and c-Fos requires ROCK activity. ROCK is a serine/threonine kinase^49^. ROCK also activates serine/threonine kinases, p38 MAPK and PKC^50^,^51^. It has been shown that both p38 MAPK and PKC induce phosphorylation of c-Fos and c-Jun *in vitro* and *in vivo*^52^,^53^. It remains to be determined whether ROCK directly or indirectly mediates c-Fos and c-Jun phosphorylation in response to matrix stiffening. Although our studies implicate a definitive role for c-Fos/c-Jun in the ‘upstream' regulation of α_6_-expression in response to matrix stiffness, the ‘downstream' effects of α_6_ induction on cellular invasiveness may involve intracellular pathways that require further study. It has been shown that α_6_-integrins activate the small GTPase RAC by a PI3K-dependent mechanism^54^. RAC activation promotes mesenchymal cell invasion into matrix barriers through mesenchymal–amoeboid transition^14^. α_6_-Integrins also activate Src family kinase^55^,^56^. Src family kinase signalling is known to promote cancer cell invasion^57^.

In this study, we found that lung (myo)fibroblasts primarily express α_6_B. Compared with α_6_A, α_6_B contains an alternative cytoplasmic domain that is 17 amino acids shorter and bears no sequence homology with α_6_A^37^. Whether the distinct cytoplasmic domain of α_6_B plays a functional role in the regulation of lung myofibroblast invasion into the BM, either by modulating myofibroblast adhesion to laminins in the BM and/or by activating cellular signals that mediate invasion is currently not known. Previous studies have shown that macrophages expressing α_6_Aβ_1_ or α_6_Bβ_1_ differ markedly in their morphology and migration on laminin matrix^58^. Macrophage adhesion to laminin matrix is regulated by phosphorylation of the cytoplasmic domain of α_6_-integrins at the serine residues^59^. It has also been reported that α_6_Aβ_1_ and α_6_Bβ_1_ differentially regulate tyrosine phosphorylation of paxillin on laminin matrix^60^.

AP-1 is a heterodimer composed of proteins belonging to the c-Fos, c-Jun, ATF and JDP families. We demonstrated that the prototypic members of AP-1 transcription factor family, c-Fos and c-Jun, mediate stiff matrix-induced α_6_-gene expression. In previous studies, Eferl *et al*.^29^ have shown that Fos-related Fra2 transgenic mice develop spontaneous fibrosis in various organs with predominant involvement of the lung. Fichtner-Feigl *et al*.^32^ have reported that the Fra2/c-Jun complex mediates IL-13/IL-13α_2_ receptor-dependent activation of the TGF-β1 promoter in bleomycin-induced mouse lung fibrosis. However, in our studies, Fra2 does not appear to be involved in matrix stiffness-regulated a6 expression. Thus, distinct AP-1 transcription factor complexes may be responsible for different components of fibrogenic signalling pathways. Since AP-1 is a heterodimer, blocking c-Fos/c-Jun with T-5224, a selective Fos inhibitor, may interrupt the function of other Fos-containing AP-1 complexes. Therefore, T-5224 treatment might not only block stiff matrix-induced α_6_-expression and myofibroblast invasion, but other potential fibrogenic signals regulated by Fos-containing AP-1 complexes.

MMPs, including MMP-2 and MMP-9 of type IV collagenases, are critical players in the pathogenesis of human IPF^61^. In this study, we demonstrated that MMP-2 is the primary type IV collagenase that mediates matrix stiffness-regulated IPF lung myofibroblast invasion into the BM. Interestingly, matrix stiffness regulates MMP-9 expression at the mRNA level, although MMP-9 activity is not detected. In addition to MMP-9, matrix stiffness also regulates mRNA expression of MMP-11, MMP-12 and MMP-16 (Table 1). AP-1 has been shown to mediate MMP expression in response to phorbol myristate acetate and cytokines^62^. Although bioinformatics analyses identified potential AP-1-binding sites in the promoter region of MMP-9, MMP-11, MMP-12 and MMP-16, we found that neither T-5224 nor AP-1 decoy oligodeoxynucleotides blocked matrix stiffness-regulated MMP-9, MMP-11, MMP-12 and MMP-16 mRNA expression (Supplementary Fig. 5e). These data suggest that matrix stiffness-regulated gene expression of MMP-9, MMP-11, MMP-12 and MMP-16, unlike the integrin α_6_ subunit, may occur via AP-1-independent mechanisms.

It is currently unclear if matrix stiffness is a cause or consequence of organ fibrosis^63^. There is accumulating evidence that mechanical interactions between myofibroblasts and stiffened matrix provide a feed-forward mechanism that maintains pro-fibrotic myofibroblast phenotypes and, therefore, perpetuation of fibrosis^1^,^3^,^4^,^5^,^6^. In rat carbon tetrachloride model of liver fibrosis, it has been observed that matrix stiffness increases before myofibroblast differentiation and fibrosis^64^,^65^. This early increase in liver stiffness can be blunted by inhibition of collagen cross-linking enzymes of the lysyl oxidase family^64^. These interesting findings suggest that changes in the mechanical properties of the ECM may not only sustain myofibroblast phenotype but contribute to the emergence of myofibroblasts in early liver fibrosis. Altogether, these studies imply that new therapies that target deleterious mechanical signals may be effective in preventing or arresting the progression of fibrosis.

In summary, the findings from this study support an essential role of the mechanosensing α_6_-integrin in mediating myofibroblast invasion and lung fibrosis following injury. Importantly, this novel mechanosensing pathway represents a target for developing new anti-fibrotic therapeutic strategies. Strategies for blocking the deleterious function of mechanosensing α_6_ may include the development of specific antibodies against fibroblast α_6_-integrins, specifically α_6_Bβ_1_ or pharmacological disruption of the mechanotransduction pathway involved in α_6_-expression. Interestingly, miR-29, an anti-fibrotic master regulator capable of blocking and reversing pulmonary fibrosis^66^,^67^, directly targets both α_6_-integrin and laminin^68^. Future studies that focus on targeting the invasive phenotype of myofibroblasts, in addition to other pro-fibrotic properties such as apoptosis-resistance, may prove to be effective in treating fibrotic disorders.

## Methods

### Lung fibroblast isolation and treatments

Human lung fibroblasts were established from tissue samples from patients undergoing lung transplantation. Previous studies have shown that lung myofibroblasts isolated from patients with IPF acquire an invasive phenotype, whereas normal human lung fibroblasts do not invade^20^. IPF lung myofibroblasts were used in this study. The studies involving human subjects were approved by institutional review board at the University of Alabama at Birmingham. Participants have been provided with written informed consent. Lung fibroblast isolation, culture, transfection, sorting and treatment were described in Supplementary Methods.

### Matrigel invasion assay

Fibrotic lung fibroblasts were cultured on soft (1 kPa) and stiff (20 kPa) PA gels for 48 h. Cells were detached from PA gels by trypsinization. An equal number of living cells (1 × 10^5^ cells per chamber) derived from soft and stiff matrix were plated in Matrigel invasion chambers (BD Biosciences, San Jose, CA, USA). Cell invasion was measured at 7 h after incubation on Matrigel to minimize the potential effect of differential matrix stiffness on fibroblast proliferation^1^. Non-invading cells at the bottom of invasion chambers were swiped with cotton swabs. Invading cells on the other side of Matrigel membrane were stained with 0.5% crystal violet for 30 min. The number of invading cells was counted under a Nikon Eclipse TE 300 microscope equipped with Spot Insight CCD camera. Invasion index was calculated as the ratio of the per cent invasion of test cells (cells cultured on soft and stiff PA gels) over the per cent invasion of control cells (cells cultured on regular tissue culture plates). In a second approach, an equal number of fibrotic lung fibroblasts were seeded on soft or stiff PA gels. Cells were allowed attachment for 1 h. Cells together with PA gels were transferred to Matrigel invasion chambers with the apical side of cells in close contact with Matrigel (Supplementary Fig. 3). Invading cells were counted at 48 h.

### Proteolytic degradation of collagen IV in the BM

Six-well Matrigel invasion chambers were incubated with DQ-collagen IV (Molecular Probes, Eugene, OR) diluted in serum-free DMEM at a final concentration of 25 μg ml^−1^ at 37 °C in dark overnight. The chambers were briefly rinsed with serum-free DMEM. Fibrotic lung fibroblasts were trypsinized from stiff matrix. In all, 1 × 10^5^ cells were seeded into each invasion chamber in the presence or absence of NKI-GoH3 (10 μg ml^−1^), α_6_-siRNA and MMP-2/MMP-9 Inhibitor I (25 μM). Cells were then incubated in a CO_2_ incubator at 37 °C for 3 h. Proteolytic degradation of DQ-collagen IV in the BM was imaged with confocal laser-scanning microscopy as described below.

### CRISPRi

Two 20-base sgRNAs were designed to target AP-1-binding TREs at −2,873/−2,879 nt and −4,848/−4,854 nt in human α_6_-promoter, respectively. AP1sgRNA1 (5′- CTAAAACTGAGTCATAAGGC -3′) binds to the plus-strand sequence at −4,841/−4,860 nt near the distal TRE1 in human α_6_-promoter. AP1sgRNA2 (5′- CACCCAACTCTGTTTTACAA -3′) binds to the minus-strand DNA at −2,924/−2,943 nt near the proximal TRE2 (Fig. 1h). Both of the sequences were cloned into pX333 (Addgene) to obtain pX333-AP1sgRNA1-AP1sgRNA2 plasmid. A DNA fragment encoding dCas9-BFP-KRAB domain was amplified by PCR from pHR-SFFV-dCas9-BFP-KRAB (Addgene). The fragment was subcloned into pX333-AP1sgRNA1-AP1sgRNA2 to obtain pX333-AP1sgRNA1-AP1sgRNA2-dCas9-BFP-KRAB plasmid. The latter plasmid and pX333 empty vector were transfected into IPF lung myofibroblasts using a Nucleofector device (Amaxa) as previously described^4^.

### Immunofluorescence and confocal laser-scanning microscopy

Eight micrometre cryostat sections were rehydrated in phosphate-buffered saline for 10 min. Tissue sections were blocked with 5% normal goat serum and co-stained with anti-αSMA (Sigma, St Louis, MO, Cat# A2547, 1:200 dilutions) and anti-α_6_ (Abcam, Cambridge, MA, Cat#14-0495, 1:300 dilutions) antibodies diluted in phosphate-buffered saline containing 1% goat serum, 0.3% Triton X-100 and 0.01% sodium azide according to manufacturer's instructions. Fluorochrome-conjugated secondary antibodies (SouthernBiotech, Birmingham, AL) were used according to the manufacturer's recommendation. Nuclei were stained with DAPI (Thermo Fisher Scientific, Waltham, MA). Fluorescent signals were detected using a confocal laser-scanning microscope Zeiss LSM710 confocal microscope equipped with a digital colour camera (Oberkochen, Germany). All fluorescent images were generated using sequential laser scanning with only the corresponding single-wavelength laser line, activated using acousto-optical tunable filters to avoid cross-detection of either one of the fluorescence channels.

### Animals and experimental protocol

The animal studies were performed in accordance with the NIH guidelines for Care and Use of Laboratory Animals. Animal usage and bleomycin protocols were approved by the Institutional Animal Care and Use Committee of the University of Alabama at Birmingham. To generate mesenchymal cell-specific *Itga6*^*−/−*^ mice, C57BL/6-*Itga6*^*fl/fl*^ mice^69^ (a gift from Dr Elisabeth Georges-Labouesse, Institut de Génétique et de Biologie Moléculaire et Cellulaire, Illkirch, France) were cross-bred with C57BL/6 mice carrying a tamoxifen-inducible Cre-recombinase (Cre-ER(T)) under the control of a regulatory sequence from procollagen I gene (The Jackson Laboratory, Bar Harbor, ME). Six- to eight-week-old female conditional *Itga6*^*−/−*^ mice and WT C57BL/6 mice were used in this study. Bleomycin sulphate (Almirall, Barcelona, Spain) was dissolved in sterile saline solution and intratracheally instilled into mice by a Stepper Repetitive Pipette (Tridak, Torrington, CT) as a single dose in 50 μl saline solution per animal (1 U kg^−1^ bodyweight). Control mice received 50 μl saline. For tamoxifen (Sigma, St Louis, MO) treatment, a dosage of 50 mg kg^−1^ bodyweight per day over 9 days or an equal volume of corn oil (vehicle for tamoxifen) was injected intraperitoneally into conditional *Itga6*^*−/−*^ mice, 5 days after bleomycin administration. For T-5224 (Apexbio, Houston, TX) treatment, a dosage of 30 mg kg^−1^ bodyweight perday or an equal volume of PVP (vehicle for T-5224) was given to WT C57BL6 mice daily by oral gavage, 10 days after bleomycin administration. Mice were killed at 21 days. Lung tissues were collected and used for histochemical and immunofluorescent analyses, micro-CT scans and isolation of lung fibroblasts.

### Statistical analysis

Statistical differences among treatment conditions were determined using one-way analysis of variance (Newman–Keuls method for multiple comparisons). Values of *P*<0.05 or *P*<0.01 were considered significant.

### Data availability

All relevant data will be made available on request and/or are included with the manuscript (as figure source data or Supplementary Information files). Additional information is detailed in the Supplementary Methods.

## Additional information

**How to cite this article:** Chen, H. *et al*. Mechanosensing by the α_6_-integrin confers an invasive fibroblast phenotype and mediates lung fibrosis. *Nat. Commun.* 7:12564 doi: 10.1038/ncomms12564 (2016).

## Supplementary Material

Supplementary Information Supplementary Figures 1-7, Supplementary Methods, Supplementary References

## Figures and Tables

**Table 1:** Matrix stiffness-regulated cell adhesion and ECM molecules.

**Gene**	**Well**	**Fold up or downregulation**
		**Stiff/soft**
*MMP9*	F04	6.4±1.6**
*ITGA6*	C10	5.3±1.1**
*CTGF*	B08	4.7±1.3**
*MMP12*	E07	3.0±1.8*
*SPP1*	G01	2.4±0.8*
*MMP16*	E11	2.1±1.1
*MMP11*	E06	−5.1±2.1**
*VCAM1*	G11	−2.6±0.6**
*ADAMTS8*	A03	−2.3±0.8
*CLEC3B*	G09	−2.0±1.2

ECM, extracellular matrix.

Results are the means±s.d. of three separate experiments; *P<0.05; **P<0.01.
