# Supplementary material for: Mechanosensing by the α6-integrin confers an invasive fibroblast phenotype and mediates lung fibrosis
Source: Nat Commun. 2016 Aug 18;7:12564. doi: 10.1038/ncomms12564 (PMC4992155; doi:10.1038/ncomms12564)
Supplement: Supplementary Information — Supplementary Figures 1-7, Supplementary Methods, Supplementary References [file ncomms12564-s1.pdf]

SUPPLEMENTARY FIGURES

<u>Supplementary Figure 1</u>

A

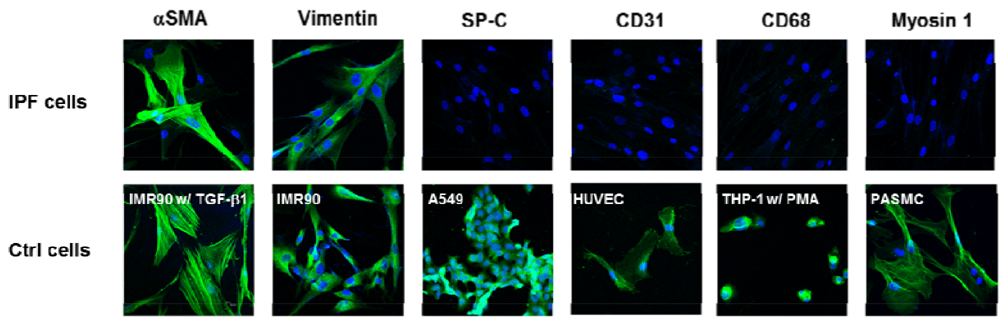

B

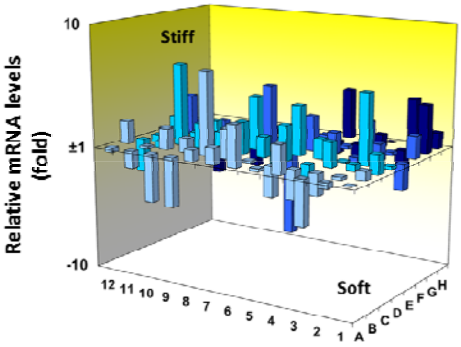

C

Gene	Well	Fold Up- or Down-Regulation (stiff/soft)	Gene	Well	Fold Up- or Down-Regulation (stiff/soft)
ITGA1	C05	1.7±0.4	ITGAL	D01	N.D.
ITGA2	C06	1.6±0.3	ITGAM	D02	N.D.
ITGA3	C07	2.1±0.9	ITGAV	D03	1.2±0.3
ITGA4	C08	1.4±0.7	ITGB1	D04	1.3±0.6
ITGA5	C09	1.7±0.5	ITGB2	D05	N.D.
ITGA6	C10	5.3±1.1**	ITGB3	D06	-1.6±0.4
ITGA7	C11	N.D.	ITGB4	D07	N.D.
ITGA8	C12	N.D.	ITGB5	D08	1.5±0.6

<u>Supplementary Figure 1</u>: Effects of matrix stiffness on relative gene transcription of cell adhesion and ECM molecules in primary lung myofibrolasts isolated from patients with IPF. **A**, Characterization of lung (myo)fibroblasts isolated from patients with IPF. Cells were stained for markers of mesenchymal cells (vimentin and αSMA), type II alveolar epithelial cells (surfactant protein C or SP-C), endothelial cells (CD31), vascular smooth muscle cells (smooth muscle myosin 1) and macrophages (CD68). TGF-β1-treated IMR90 cells, IMR90 cells, A549, HUVEC, PMA-treated THP-1 and PASMC were using as positive controls for expression of each of markers as indicated; scale bars = 50 μm. **B**, A complete list of 84 cell adhesion and ECM genes is available at www.sabiosciences.com, array code PAHS-013Z. **C**, Up- or down-regulation of 16 integrin subunits on stiff *vs*. soft matrix are shown. Results are the means ± SD of four independent arrays; ***p* < 0.01, N.D. = not detected.

Supplementary Figure 2

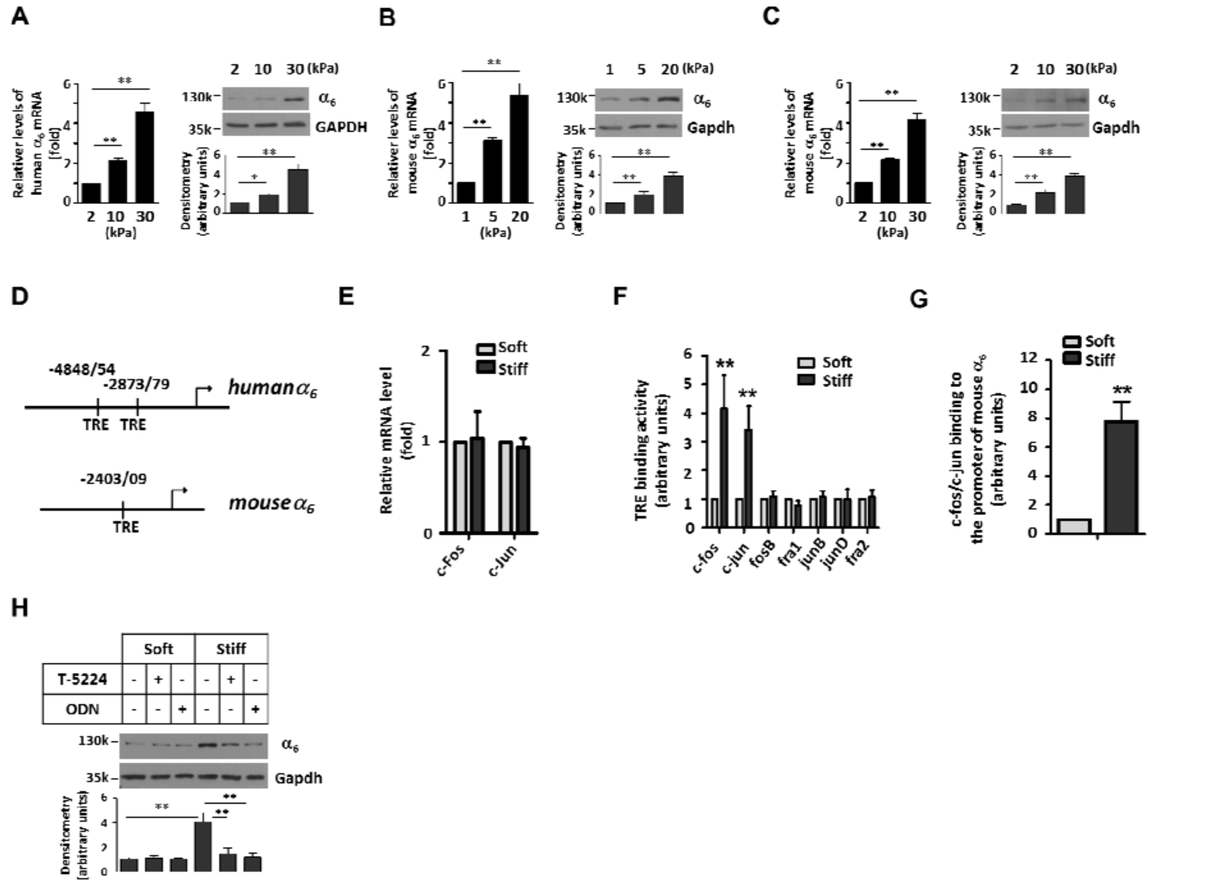

Supplementary Figure 2: Stiff matrix upregulates α_6 expression by a c-Fos/c-Jun-dependent mechanism in human and mouse lung myofibroblasts. **A**, Stiff matrix promotes α_6 mRNA and protein expression in human IPF lung myofibroblasts cultured on PDMS hydrogels with increasing stiffness (2, 10 and 30 kPa). Levels of α_6 mRNA and protein were determined by qPCR and immunoblot analysis, respectively. **B**, Stiff matrix promotes α_6 mRNA and protein expression in primary mouse lung myofibroblasts cultured on PA gels with increasing stiffness (1, 5 and 20 kPa). **C**, Stiff matrix promotes α_6 mRNA and protein expression in primary mouse lung myofibroblasts cultured on PDMS hydrogels with increasing stiffness (2, 10 and 30 kPa). **D**, Schematics depict AP-1-binding TREs in the promoter regions of human and mouse α_6. **E**, Effects of matrix stiffness on c-Fos and c-Jun mRNA expression were evaluated by qPCR analysis. **F**, Nuclear extracts derived from mouse lung myofibroblasts cultured on soft or stiff PA gels were incubated with immobilized oligonucleotides containing

TREs. The TRE binding activities of c-fos, c-jun, fosB, fra1, junB, junD and fra2 were quantified by colorimetric ELISA. **G**, The binding of c-fos/c-jun complex to mouse α_6 promoter under soft *vs.* stiff matrix conditions was measured by quantitative ChIP. **H,** Effects of c-fos/c-jun inhibitors (T-5224 and decoy ODNs) on matrix stiffness-regulated mouse α_6 expression were evaluated by immunoblot. Results are the means $\pm$ SD of at least three separate experiments; *$p < 0.05$; **$p < 0.01$.

Supplementary Figure 3

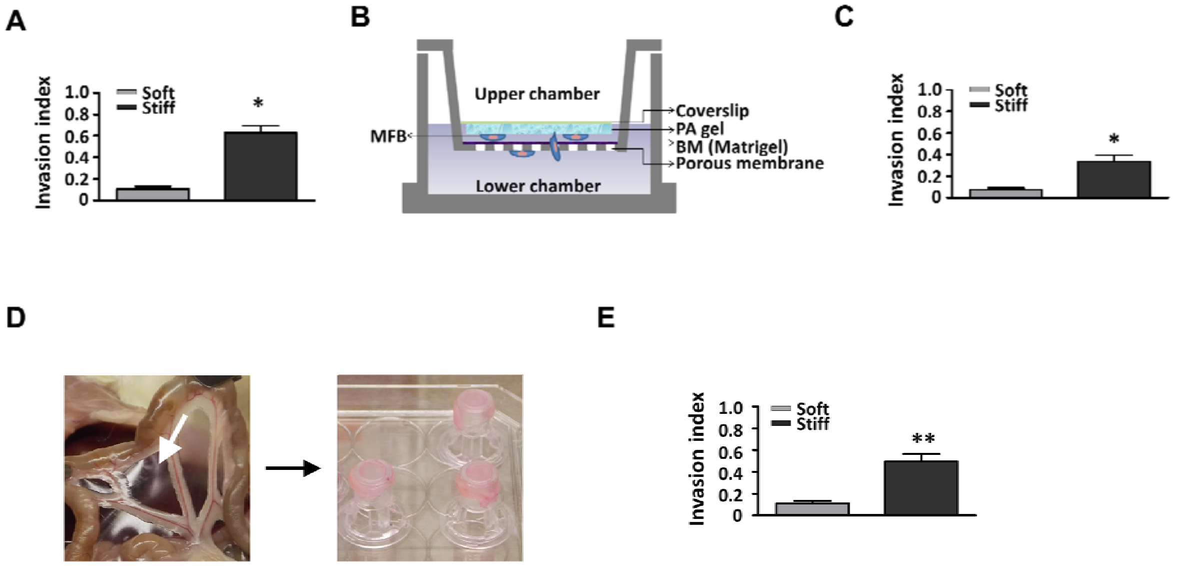

Supplementary Figure 3: Evaluation of matrix stiffness-regulated lung myofibroblast invasion into the BM by PDMS hydrogel system, a self-developed "Sandwich" system and rat mesentery. **A**, IPF lung myofibroblasts were adapted to soft (2 kPa) and stiff (30 kPa) PDMS gels. Cells were detached by trypsinization. The ability of IPF myofibroblasts cultured on soft vs. stiff PDMS gels to invade the BM was evaluated by Matrigel invasion assay. **B**, Schematic shows a "sandwich" invasion assay system. **C**, Invasion indexes in the "sandwich" invasion assay were calculated as the ratio of the percent invasion of test cells (lung MFBs cultured on soft or stiff gels) over the percent invasion of control cells (lung MFBs cultured on the regular tissue culture plates). **D**, Rat mesentery (white arrow) was isolated from euthanized rats, decellularized by 0.5 N ammonium hydroxide and mounted on 24-well invasion inserts using Super Glue. **E**, An equal number of living IPF lung myofibroblasts pre-cultured on soft or stiff PA gels were plated on rat mesentery for 24 hours. Invading cells in the lower chambers

were detached by trypsinization and the number of cells was counted. Invasion indexes were calculated as described in C. Results are the means ± SD of at least three independent arrays; **p* < 0.05. ***p* < 0.01.

Supplementary Figure 4

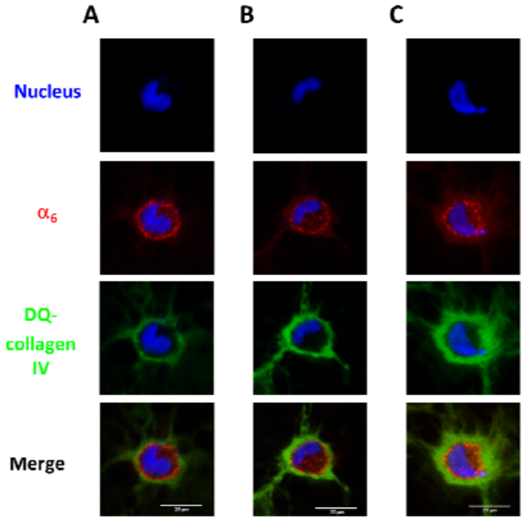

Supplementary Figure 4: Effects of control IgG, PVP and scrambled siRNA on proteolytic activation of DQ-collagen IV. A, control IgG; **B**, PVP; **C,** scrambled siRNA.

Supplementary Figure 5

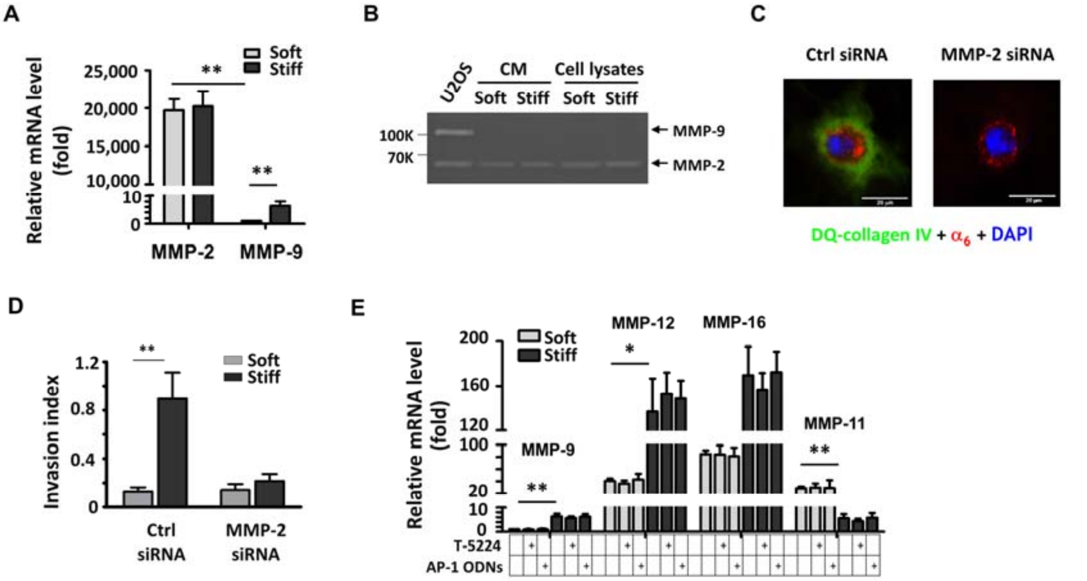

Supplementary Figure 5: MMP-2 mediates matrix stiffness-regulated lung myofibroblast invasion into the BM. **A**, Relative mRNA levels of MMP-2 and MMP-9 in IPF lung myofibroblasts cultured on soft and stiff PA gels were evaluated by qPCR. **B**, Zymographic analysis of MMP-2 and MMP-9 activities in cell lysates and the conditioned media (CM) collected from IPF lung myofibroblasts cultured on soft *vs.* stiff PA gels. CM from U2OS cells were used as positive control for MMP-2 and MMP-9 activities. Loading volumes of CM and cell lysates were normalized to equal cell numbers under soft and stiff matrix conditions. **C**, MMP-2-specific siRNA or control siRNA was transfected into IPF lung myofibroblasts. Pericellular proteolysis of DQ-collagen IV in MMP-2-deficient lung myofibroblasts was determined by confocal immunofluorescent microscopy. α_6 expression was stained red. Nuclei were stained blue. **D**, IPF lung myofibroblasts transfected with MMP-2 siRNA and control siRNA were pre-cultured on soft and stiff PA gels. The ability of IPF myofibroblasts cultured on soft *vs.* stiff matrix to invade the BM was evaluated by Matrigel invasion assay. **E**, Relative mRNA levels of MMP-9, MMP-12, MMP-16 and MMP-11 in IPF lung myofibroblasts cultured on soft and stiff PA gels in the presence or absence of T-5224 or AP-1 ODNs were evaluated by qPCR. Results are the means ± SD of at least three separate experiments; $^*p < 0.05$, $^{**}p < 0.01$.

Supplementary Figure 6

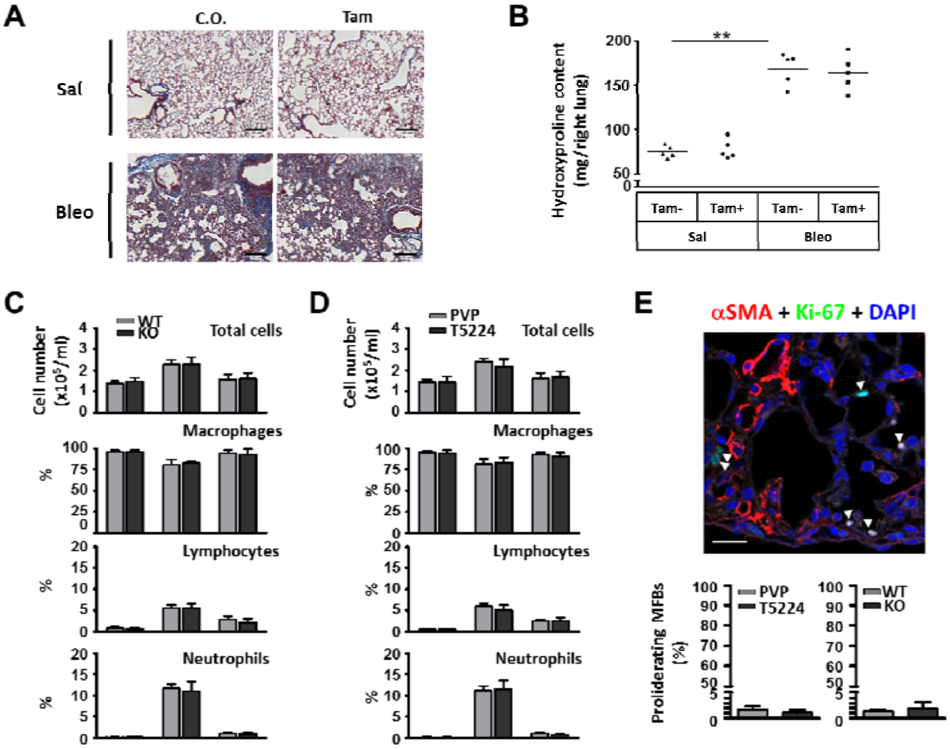

Supplementary Figure 6: **A** and **B**, Comparisons of bleomycin-induced lung fibrosis in *Itga6* floxed mice treated with tamoxifen *vs.* corn oil (C.O.) by trichrome stain (**A**) and hydroxyproline content assay (**B**). **C** and **D**, Quantification of the numbers of total cells, macrophages, lymphocytes and neutrophils in BAL fluids at day 14 in WT, α_6 KO, PVP-treated and T-5224-treated mice (n = 5/group). **E**, Confocal immunofluorescent microscopy shows Ki-67 expression (green) and αSMA-positive lung myofibroblasts (red) in mice treated with bleomycin. Nuclei were stained by DAPI (blue); scale bar = 20 µm. Results are the means $\pm$ SD of at least three separate experiments; **p < 0.01

Supplementary Figure 7

Figure 1A

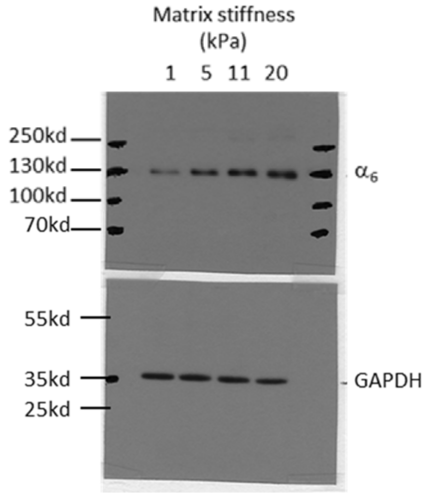

Figure 1D

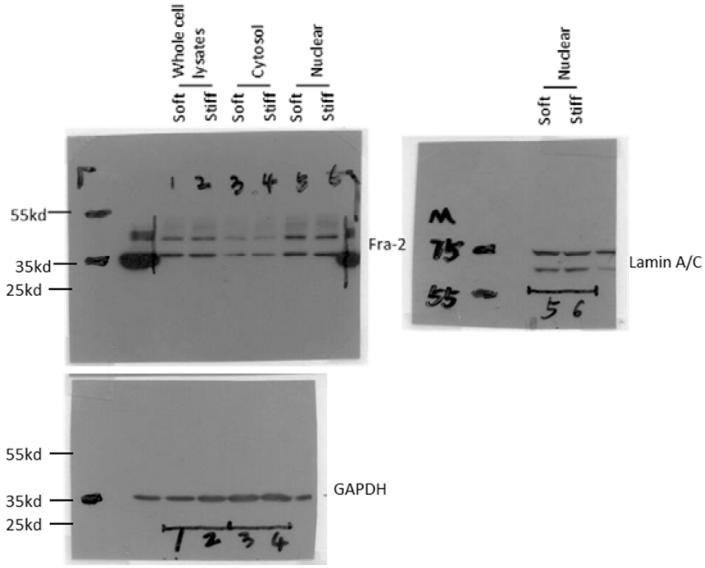

Figure 1E

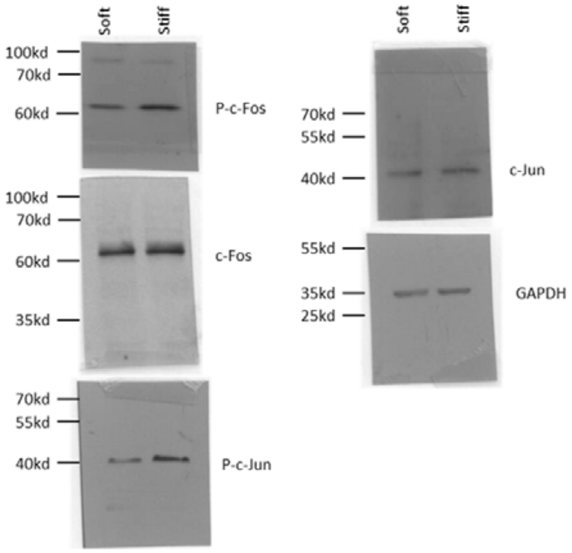

Figure 1F

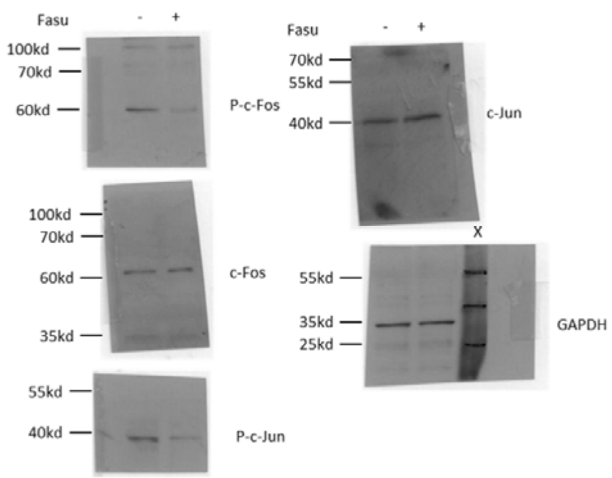

Figure 1F (cont'd)

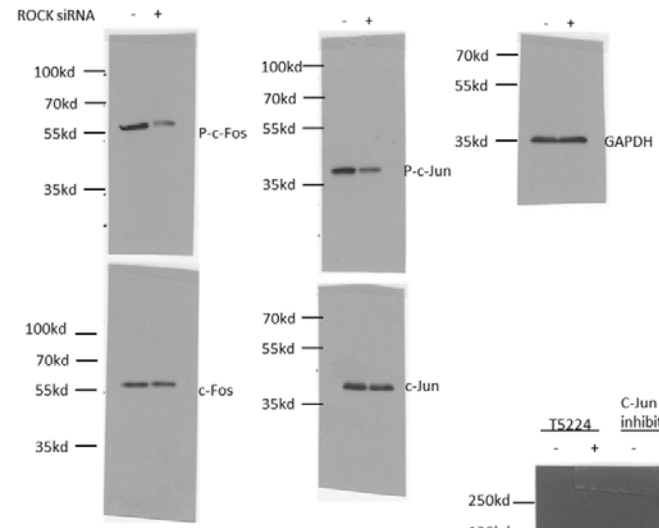

Figure 1H

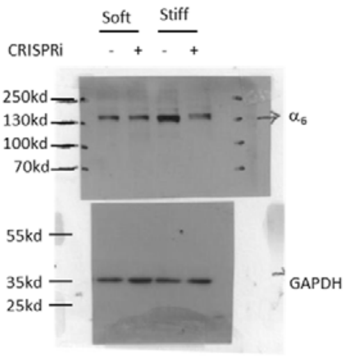

Figure 1I

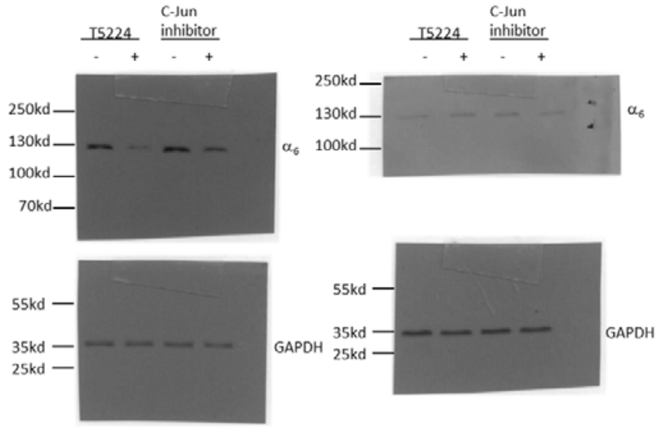

Figure 2D

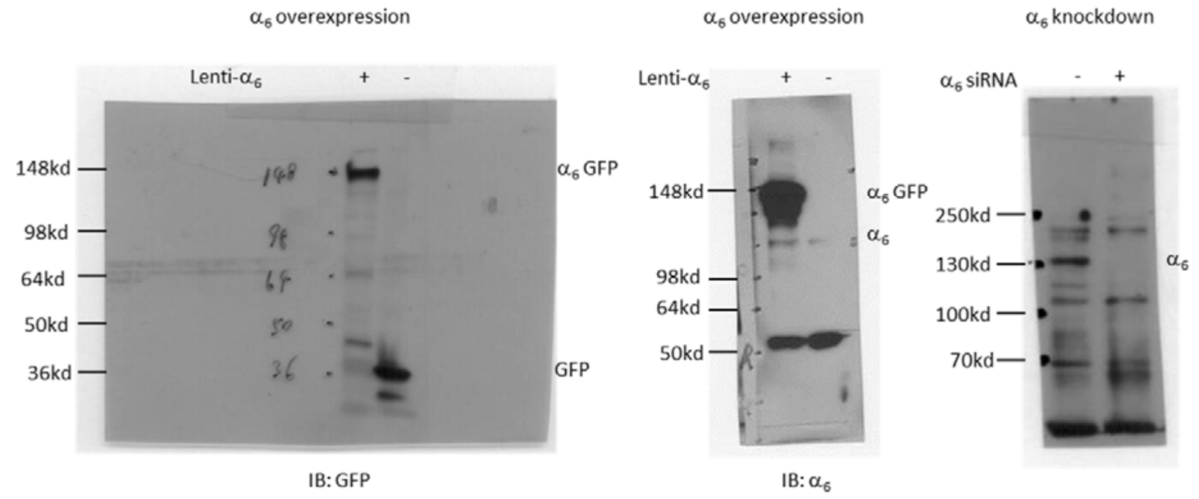

Figure 3B

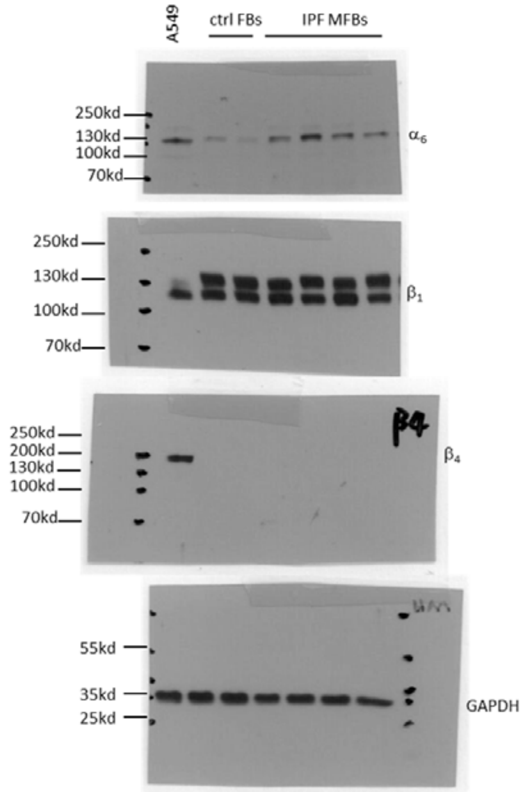

Figure 3C

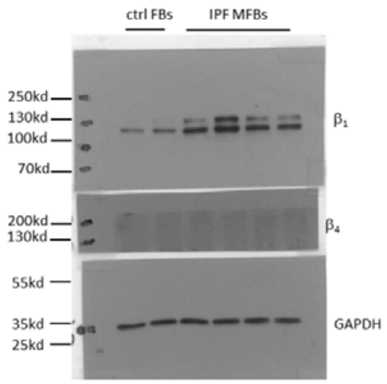

Figure 3D

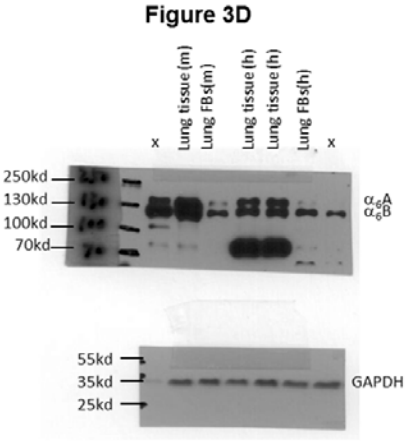

Figure 4A

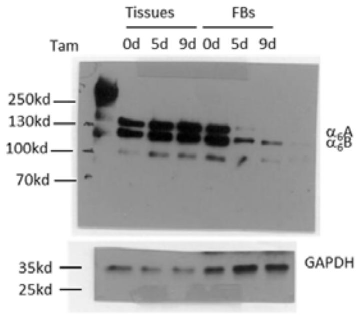

Figure 4F

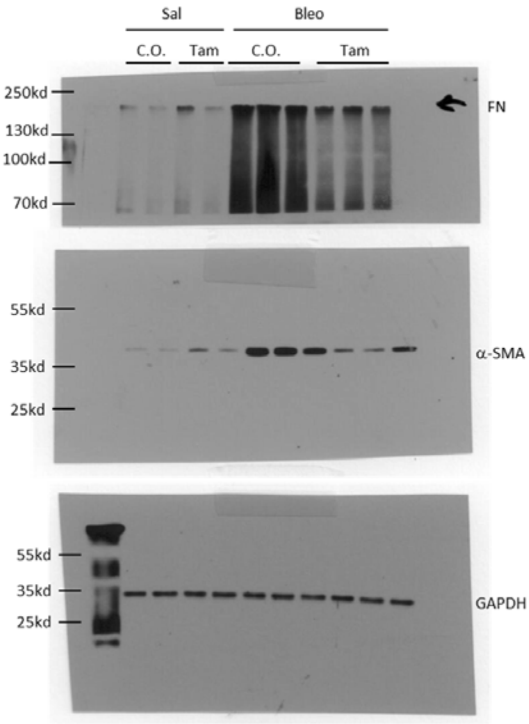

Figure 5E

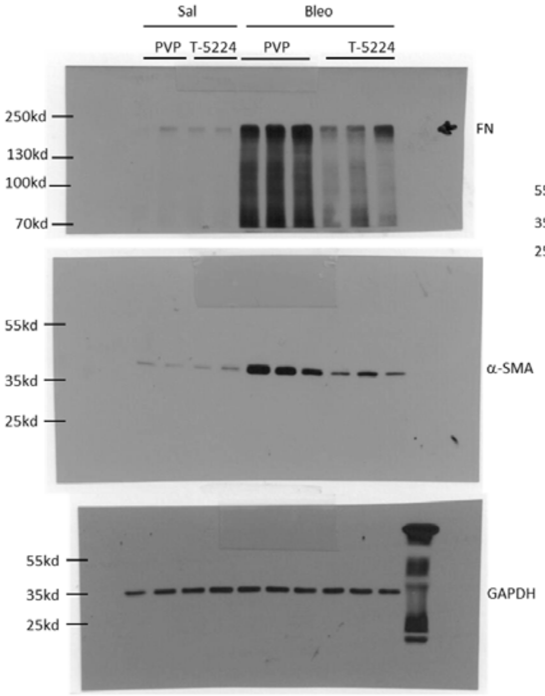

Supplementary Figure 2A

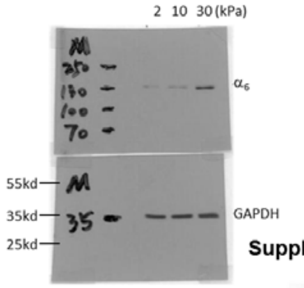

Supplementary Figure 2B

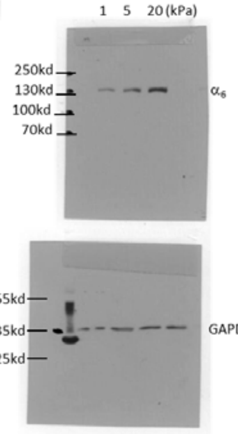

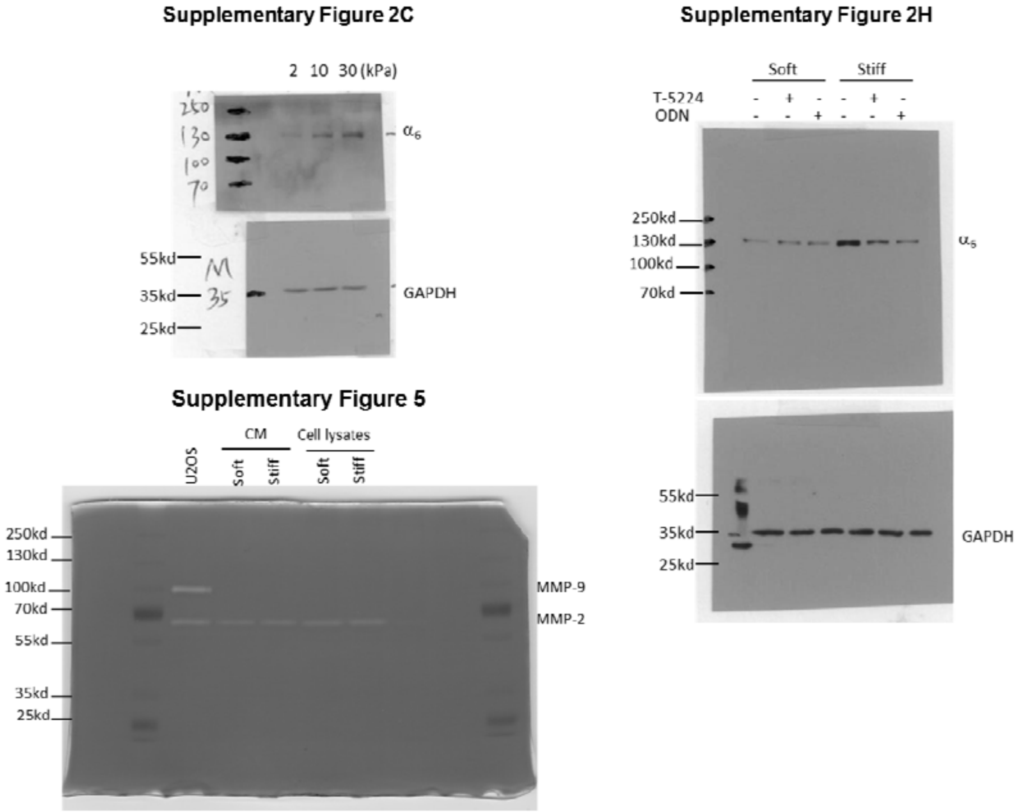

<u>Supplementary Figure 7</u>: Uncropped original immunoblots.

<u>SUPPLEMENTARY METHODS</u>

Antibodies and reagents

Anti-αSMA antibodies were purchased from Sigma (St. Louis, MO) and Thermo Scientific Pierce (Waltham, MA). Anti-α_6, -vimentin, -Prosurfactant Protein C (SP-C), -CD31, -CD68, myosin and -GFP antibodies were from Abcam (Cambridge, MA). NKI-GoH3 was from EMD Millipore (Billerica, MA). Anti-β_1, anti-β_4, anti-phospho c-Fos, anti-total c-Fos, anti-phospho c-Jun, anti-total c-Jun and anti-Ki-67 antibodies were from Cell Signaling (Danvers, MA). Anti-GAPDH antibody was from Santa Cruz Biotechnology (Santa Cruz, CA). RT^2 Profiler PCR

Array for Human Extracellular Matrix and Adhesion Molecules was purchased from Qiagen (Venlo, Netherlands). DQ-collagen IV was from Life Technologies (Grand Island, NY). α_6 siRNA was from Dharmacon (Lafayette, CO). TransAM AP-1 transcription factor ELISA kit was from Active Motif (Carlsbad, CA). T-5224 was from Apexbio (Houston, TX). c-Jun peptide inhibitor was from Tocris (Minneapolis, MN). PDMS hydrogels with 2 kPa, 10 kPa and 30 kPa were purchased from ExCellness Biotech SA (Lausanne, Switzerland).

Lung fibroblast isolation

Lungs were minced in sterile phosphate-buffered saline and tissue pieces were placed in 100-mm tissue culture dishes containing Dulbecco's modified Eagle's medium (DMEM), 10% fetal bovine serum (FBS), 1% penicillin/streptomycin/glutamine, nonessential amino acids, and sodium pyruvate (supplemented DMEM). Medium was replenished every 3 d. After 14 d, cells growing out of the explants were trypsinized and plated in supplemented DMEM. Lung fibroblasts were used between passages 6 and 10.

Preparation of polyarylamide hydrogels with varying stiffness and mechanical testing

PA gels with tunable stiffness were fabricated using a published protocol [1]. Gel surfaces were coated with 0.1 mg/ml rat tail collagen I (BD Biosciences). PA gel mechanical properties were measured using an MFP-3D-BIO Atomic Force Microscope (AFM) (Asylum Research; Santa Barbara, CA) in contact mode. Samples were probed with a 4.74 µm diameter beaded-tip (Bruker; Camarillo, CA), and cantilever spring constants were measured prior to sample analysis using the thermal fluctuation method [2], with nominal values of 20-30 mN/m. Young’s modulus

was obtained from force-indentation profiles using a Hertzian model and sample Poisson's ratio of 0.5. A minimum of 20 independent measurements per gel were obtained and analyzed.

Quantitative real-time PCR

Total RNA was isolated using TRIzol reagent (Invitrogen). 1 μg total RNA was reversely transcribed into cDNA with a cDNA Synthesis Kit (Thermo Scientific). Quantitative PCR reactions were carried out in a Bio-Rad iCycler. Relative quantification was calculated using the comparative C_T method[3].

Quantitative chromatin immunoprecipitation (ChIP) assay

Lung fibroblasts were treated with 1% formaldehyde for 10 minutes at 37°C to cross-link histones to DNA. The cross-linked chromatin was sonicated to shear into chromatin fragments of 200–1,000 base pairs. A portion of sheared chromatin was reversed at 65°C for 4hr and crosslinked DNA was purified by phenol/chloroform extraction. The DNA was saved and used for internal reference control in the following Real-time PCR reactions. The rest of sonicated chromatin was immunoprecipitated with anti-phospho c-Fos antibody at a concentration recommended by the manufacturer, while negative control was immunoprecipitated with IgG. Immunocomplexes were recovered with Protein A agarose beads. Crosslinks were reversed and treated by Proteinase K to remove protein from the DNA. DNA was purified by phenol/chloroform extraction. Real-time PCR was performed to quantify c-Fos/c-Jun-binding α_6 promoter fragments using the following primers: forward 5'- TGTACTCCCTCCCCAGGTCT-3', reverse 5'-CTGGATCCACTGAGGACACAT-3' for the proximal TRE region (-2873 ~ -2879 nt); forward 5'-TGAAGGTGGGGATGGAAAACC-3', reverse 5'-

GCCAGGAAGCCATATTTTGCC-3' for the distal TRE region (-4848 ~ -4854 nt) in the human α_6 promoter; and forward 5'- TTCAAGGCCAGATTTCCATAGG-3', reverse 5'-TGTTGTGCCTGTCTGTCTTC-3' for the TRE region (-2403 ~ -2409 nt) in the mouse α_6 promoter.

Promoter activity assay

4 μg of WT and mutated α_6 promoter constructs were co-transfected with 50 ng of Renilla luciferase-expressing control vector (pRL-CMV) (Promega) into 1x 10^6 lung myofibroblasts. Both of thymines (Ts) in WT TREs (TGAG/CTCA) in the α_6 promoter were substituted by guanines (Gs) (GGAG/CGCA) to destroy the binding of c-Fos/c-Jun transcription factor complex[4]. Transfected cells were cultured for 48 hrs. Cells were lysed with 1x passive lysis buffer (Promega). Luciferase activities were determined using a Dual-Luciferase reporter assay system (Promega). The relative light units were measured by an Orion Microplate Luminometer (Berthold, Germany). The firefly luciferase activity corresponding to a specific α_6 promoter construct was normalized to the renilla luciferase activity of the same sample. Results are expressed as fold changes compared with the mean firefly/renilla ratio of the cells cultured on soft gels taken as a unit.

Construction of α_6-expressing lentiviruses and cell infection

The full-length cDNA of human α_6 corresponding to NCBI Reference Sequence: NM_000210.2 was amplified by PCR with a pair of primers (5'-AAGCTAGCC ACCATGGCCGCCGCCGGGCAGCTGT-3' and 5'-GGACCGGTGCATCAGAAGTAAG CCTCTC-3') and subsequently cloned into a lentiviral expression vector, pLJM1-EGFP

(Addgene, Cambridge, MA). Viruses were packaged in 293T cells by co-transfection with lentiviral helper plasmids psPAX2 and pMD2.G (both from Addgene). Virus-containing conditioned medium was harvested 72 hrs after transfection, filtered, and used to infect recipient lung fibroblasts in the presence of 8 μg/mL polybrene (Sigma). Infected cells were selected with 2 μg/mL puromycin (Sigma).

siRNA-mediated gene knockdown

4 x 10^5 cells/well (6-well plates) were cultured with 0.5 mL of OptiMEM media (Life Technologies) containing 5 μL of Lipofectamine 2000 (Life Technologies) and 50 nM α_6-specific siRNA, 200 nM ROCK1- and ROCK2-specific siRNAs, 100 nM MMP-2-specific siRNA or scrambled control siRNA (Dharmacon) for 6 h. OptiMEM media were removed, and cells were cultured in 2 ml DMEM supplemented with 10% FBS for 96 h before harvesting.

Assessment of lung inflammation and injury

Mice were sacrificed and lungs were flushed with three 1mL aliquots of complete media. Recovered BAL fluid samples were centrifuged (300 g for 8 min). The cell pellets were resuspended in 0.5 ml complete media. Total cells were counted with a hemocytometer. For differential cell counts, cells were stained with Diff-Quik (Baxter Healthcare, Miami, FL) according to manufacturer's recommendation. BAL cell differential counts were determined based on standard morphological criteria and staining properties under a light microscope with evaluation of ≥500 cells/slide.

Subcellular fractionation, co-immunoprecipitation, immunoblot and densitometry analysis

Nuclear proteins and cytoplasmic proteins were isolated using NE-PER Nuclear and Cytoplasmic Extraction Kit (Thermo Scientific) as described in our previous studies[5].

Cell lysates were precleared by adding 30 μl of protein G-Sepharose (Amersham Pharmacia Biotech). Supernatants were incubated with rabbit polyclonal antibodies against α_6 at a concentration recommended by the manufacturer or an equal amount of non-immune rabbit IgG (SouthernBiotech) at 4°C for 1 h. Precipitation of the immunocomplexes was achieved by adding goat anti-rabbit IgG (SouthernBiotech) at a final concentration of 1 μg/ml. Immunocomplexes were pelleted with 100 μl of protein G-Sepharose. After 1 h of incubation at 4°C, the resins were washed three times and α_6-binding immunocomplexes were eluted by boiling in Laemmli buffer (Bio-Rad).

Immunoblot was performed as described in our previous studies[6]. Blot images were scanned. Bands were quantified by ImageJ (NIH, Bethesda).

Flow cytometry

Single cell suspensions ($1x10^6$ cells) were pelleted and washed once with wash buffer (PBS with 2% FBS and 0.1% NaN3), and then incubated with fluorescein isothiocyanate (FITC)-conjugated (direct) or unlabeled (indirect) anti-human α_6 antibody or non-immune isotype control IgG diluted in blocking buffer at a final concentration of 10 μg/ml for 60 min at 4°C. After washing 4 times with wash buffer, cells were incubated with FITC-conjugated secondary antibody (indirect only) for 60 min at 4°C. After washing 4 times with wash buffer, stained cells were fixed in PBS containing 1% paraformaldehyde. Flow cytometry was performed on a LSRII Flow Cytometer (BD Biosciences), and data were processed using FACSDiva software (BD Biosciences).

Gelatin Zymography

Conditioned media (CM) and cell lysates from IPF myofibroblasts cultured on soft and stiff matrix were resolved under denaturing and non-reducing conditions on 10% SDS-PAGE containing 1 mg/ml gelatin. CM from U2OS cells was loaded and used as MMP-2 and MMP-9 positive controls. After electrophoresis, the gels were washed twice for 15 minutes with 2.5% Triton X-100 and incubated overnight at 37° C in 50 mM Tris-HCl, 200 mM NaCl, 10 mM $CaCl_2$, pH 7.4. The gels were then stained 30 minutes with 30% methanol/10% acetic acid containing 0.5% Coomassie Brilliant Blue R-250 and destained in the same solution without dye until clear bands representing areas of gelatinolysis on the blue background were observed. Digestion bands were quantitated by an image analyzer system with GelDoc 2000 and ImageJ (NIH, Bethesda).

Lung histology and hydroxyproline content assay

Masson's trichrome stain for collagen and H&E stain were performed using a kit from Poly Scientific (Bay Shore, NY) according to the manufacturer's recommendations. Images were obtained with a Nikon Eclipse TE 300 microscope equipped with Spot Insight CCD camera and MetaMorph software version 6.2 r4 (Universal Imaging Corp., Downington, PA).

Mouse right lungs were homogenized in 2.0 ml distilled water and incubated with 125 µl of 50% trichloroacetic acid on ice for 20 min. Samples were centrifuged and the pellets were mixed with 1 ml 12 N hydrochloric acid and baked at 110°C for 14-18 h. Dry samples were dissolved in 2 ml deionized water. 200 µl samples (or standards) were added to 500 µl 1.4% chloramine T in 0.5 M sodium acetate/10% isopropanol (Fisher Scientific, Pittsburgh, PA) and incubated for 20 min at room temperature. 500 µl Ehrlich's solution (1.0 M p-dimethylaminobenzaldehyde in 70% isopropanol/30% perchloric acid) (Fisher Scientific) was added, mixed, and incubated at

65°C for 15 min. Optical density of each sample and standard was measured at 550 nm and the concentration of lung hydroxyproline was calculated from a hydroxyproline standard curve.

Micro-computed tomography (Micro-CT) and 3D volume reconstructions

Mouse lungs were inflated with 4% paraformaldehyde in PBS and fixed in fresh fixative for 4 h. Fixed lungs were transferred to 15% sucrose in PBS and left overnight at 4°C. Lungs were dehydrated for 2 h each in 70%, 80% and 90% ethanol and then overnight in 100% ethanol. Dehydrated lungs were incubated in 100% hexamethyldisilazane for 2 h. After air dry, lungs were placed in a 16 mm diameter sample holder and scanned using a Scanco μCT40 scanner (SCANCO Medical, AG, Switzerland) with the following settings: 45 kVp, 177 μA and an integration time of 200 ms. Scans were automatically reconstructed into 2D slices with an element size of 16 μm in all three spatial dimensions. Using the μCT Evaluation Program (V6.5-2, Scanco Medical), lungs were outlined to include all lung tissues but excluding the trachea. 3D analyses were performed on the outlined lungs. A fixed threshold of 32 (of maximal grey-scale value) was used to determine the whole lung volumes. A further analysis using a fixed threshold of 120 was used to determine the lung tissue volumes. Aerated lung volumes were calculated by subtraction of the lung tissue volumes from the whole lung volumes.

SUPPLEMENTARY REFERENCES

1. Tse, J.R. & Engler, A.J. Preparation of hydrogel substrates with tunable mechanical properties. *Curr Protoc Cell Biol* **Chapter 10**, Unit 10 16 (2010).
2. Levy, R. & Maaloum, M. Measuring the spring constant of atomic force microscope cantilevers: thermal fluctuations and other methods. *Nanotechnology* **13**, 33-37 (2002).
3. Livak, K.J. & Schmittgen, T.D. Analysis of relative gene expression data using real-time quantitative PCR and the 2(-Delta Delta C(T)) Method. *Methods (San Diego, Calif* **25**, 402-408 (2001).

4. Huang, X., *et al.* Matrix Stiffness-Induced Myofibroblast Differentiation Is Mediated by Intrinsic Mechanotransduction. *Am J Respir Cell Mol Biol* (2012).
5. Zhou, Y., Hagood, J.S., Lu, B., Merryman, W.D. & Murphy-Ullrich, J.E. Thy-1-integrin alphav beta5 interactions inhibit lung fibroblast contraction-induced latent transforming growth factor-beta1 activation and myofibroblast differentiation. *J Biol Chem* **285**, 22382-22393 (2010).
